# Analysis of telomere-to-telomere genome of red carrot TXH4 elucidates the role of DcLCYE and DcLCYB1 in lycopene accumulation in carrot

**DOI:** 10.1093/hr/uhaf192

**Published:** 2025-07-29

**Authors:** Xiao-Jie Li, Yong-Chao Hao, Jun-Wei Zheng, Ya-Hui Wang, Jia-Xing Tian, Chen-Hao Zhang, Cong-Sheng Yan, Lin Zhou, Xiao-Ming Song, Ai-Sheng Xiong, Yi Liang

**Affiliations:** National Engineering Research Center for Vegetables, Beijing Vegetable Research Center, Beijing Academy of Agriculture and Forestry Sciences, Beijing 100097, China; Beijing Key Laboratory of Vegetable Germplasm Improvement, Beijing Academy of Agriculture and Forestry Sciences, Beijing 100097, China; State Key Laboratory of Wheat Improvement, College of Agronomy, Shandong Agricultural University, Tai'an, Shandong 271018, China; Institute of Agricultural Science and Technology of Zhengzhou, Potato Research Institute, Zhengzhou 450015, China; State Key Laboratory of Crop Genetics & Germplasm Enhancement and Utilization, Ministry of Agriculture and Rural Affairs Key Laboratory of Biology and Germplasm Enhancement of Horticultural Crops in East China, College of Horticulture, Nanjing Agricultural University, Nanjing, Jiangsu 210095, China; National Engineering Research Center for Vegetables, Beijing Vegetable Research Center, Beijing Academy of Agriculture and Forestry Sciences, Beijing 100097, China; Beijing Key Laboratory of Vegetable Germplasm Improvement, Beijing Academy of Agriculture and Forestry Sciences, Beijing 100097, China; School of Life Sciences, North China University of Science and Technology, Tangshan 063210, China; Institute of Vegetables Research, Anhui Academy of Agricultural Sciences, Hefei 230001, China; College of Plant Protection, Henan Agricultural University, Zhengzhou 450046, Henan, China; School of Life Sciences, North China University of Science and Technology, Tangshan 063210, China; State Key Laboratory of Crop Genetics & Germplasm Enhancement and Utilization, Ministry of Agriculture and Rural Affairs Key Laboratory of Biology and Germplasm Enhancement of Horticultural Crops in East China, College of Horticulture, Nanjing Agricultural University, Nanjing, Jiangsu 210095, China; National Engineering Research Center for Vegetables, Beijing Vegetable Research Center, Beijing Academy of Agriculture and Forestry Sciences, Beijing 100097, China; Beijing Key Laboratory of Vegetable Germplasm Improvement, Beijing Academy of Agriculture and Forestry Sciences, Beijing 100097, China

## Abstract

Carrot taproots exhibit a wide range of colors due to variations in carotenoid and anthocyanin contents. TouXinHong4 (TXH4), a Chinese red carrot landrace from western China, is appreciated for its storability, stress tolerance, and good flavor. In this study, we generated a high-quality, telomere-to-telomere (T2T), gap-free genome assembly of TXH4, with a total size of 449.92 Mb. Repetitive sequences accounted for 48.6% of the genome. A total of 34 225 genes were identified, with 34 016 genes associated with at least one functional annotation. Comparison with two previously assembled carrot genomes, *Daucus carota* T2T (DcT2T) and *D. carota* v2.0 (DcRef), revealed 2 466 422 and 2 037 986 single nucleotide polymorphisms and 500 579 and 474 704 insertions/deletions in DcT2T and DcRef, respectively. Carotenoid analysis showed that the lycopene content in TXH4 roots was 1965-fold higher than that in the leaves, while α-carotene and β-carotene levels in the roots were only 2.7% and 3.5% of those in the leaves, respectively. This finding was consistent with the lack of transcription of *lycopene β-cyclase 1* (*LCYB1*) and *lycopene ε-cyclase* (*LCYE*) in TXH4 roots. Furthermore, overexpression of *DcLCYB1* and *DcLCYE* resulted in reduced lycopene levels, while their knockout led to elevated lycopene accumulation. Downregulation of *DcLCYB1* and *DcLCYE* was identified as a critical factor contributing to lycopene accumulation, resulting in the red root phenotype of TXH4 roots. The gapless genome assembly of TXH4 offers important insights into the red carrot genome and expands the genomic resources for breeding, facilitating more efficient genome-assisted breeding strategies for crop improvement.

## Introduction

Cultivated carrot (*Daucus carota* var. *sativus*, 2*n* = 18) is believed to have emerged in Central Asia and classified into two major groups: western and eastern [1]. The wide genetic diversity found in carrot germplasm, including landraces, cultivars, and wild carrots, forms the foundation for modern carrot breeding. Carrot germplasm displays a variety of colors, including white, yellow, orange, red, and purple. Root color is a key trait in breeding programs and a significant marker of the carrot’s breeding history [2, 3].

Wild carrots typically produce white roots due to the absence of carotenoid and anthocyanin pigments [4]. During the initial stages of domestication, carrot roots were not orange but rather yellow or purple [5, 6]. It was not until after 1600 that orange carrots began to be depicted in paintings [7]. The preference of breeders for orange carrots led to an increased accumulation of vitamin A carotenoids, such as α-carotene and β-carotene, in cultivated varieties [8]. Red carrots first appeared in China and India around the 1700s and remain commercially important of Asia [7, 9, 10]. In China, red carrots are primarily cultivated in western regions, including Xinjiang, Gansu, and Shanxi provinces. These red carrots are predominantly landraces, which tend to have lower levels of genetic diversity and heterozygosity. However, the available genetic information on red carrots remains limited. A high-quality genome of the red carrot might help reveal the phylogenetic relationship between Chinese orange and red carrots and further advance carrot breeding research.

A telomere-to-telomere (T2T) genome refers to a complete, high-quality sequence without gaps, covering all centromeric and repetitive regions. This level of completeness is enabled by ultra-long read sequencing technology [11]. With advancements in sequencing platforms, several plant T2T genomes have been released, including those of Arabidopsis [12], watermelon [13], bitter melon [14], cassava [15], strawberry [16], wild blueberry [17], melon [18, 19], and pumpkin [20]. In 2023, the first T2T genome of orange carrot ‘Kurodagosun’ was assembled and released. Although derived from a highly inbred line, the carrot’s outcrossing nature results in a relatively high heterozygosity rate of 0.6% in ‘Kurodagosun’ [21]. An improved assembly of the orange, Nantes-type, doubled-haploid carrot genome DH1, along with the resequencing of a large number of carrot accessions, has offered deeper insights into the domestication and breeding advancements of carrots. However, the DH1 genome still covered only 93% of the genome size [22]. More recently, a T2T gapless assembly of another Nantes-type orange carrot variety, DH13M14, was published in 2024 [23]. To date, however, no high-quality reference genome has been available for red carrot.

Carrot serves as a model system to analyze carotenoids [24–27]. The regulatory patterns underlying carotenoid accumulation in red carrot are distinct from those in orange carrot [28]. The selection of orange carrots led to increased levels of provitamin A carotenoids, including α-carotene and β-carotene, in cultivated carrots. These compounds are produced from lycopene through the catalytic actions of lycopene β-cyclase (LCYB) and lycopene ε-cyclase (LCYE). *LCYB* expression directs lycopene towards the β-branch of the carotenoid synthesis pathway, while the production of α-carotene warrants the combined activities of both LCYE and LCYB. Previous studies have demonstrated that *LCYB* expression in tomato fruits effectively transforms lycopene into β-carotene [29]. *ClLCYB* overexpression in watermelon has been shown to reduce lycopene accumulation and increase β-carotene accumulation, resulting in the flesh color changing from red to orange. Conversely, *ClLCYB* downregulation significantly increased lycopene levels, generating red-fleshed watermelons from the varieties that originally yielded pale-flesh fruits [30]. In bananas, the CRISPR/Cas9-edited lines that targeted *LCYE* exhibited a drastic decrease in α-carotene levels, with lycopene being directed into β-carotene biosynthesis [31]. The roots of red carrot varieties Benhongjinshi, Meiguihong, Dayumeirenzhi, and Betafruit exhibit notably lower *DcLCYE* expression than orange carrot roots, resulting in a higher lycopene accumulation. *DcLCYE* overexpression in red carrots causes lycopene to be channeled into the α-carotene biosynthesis branch, shifting the root color from red to orange or yellow [32]. However, the role of DcLCYB in carotene accumulation in carrots remains unclear.

In this study, we presented a complete T2T genome assembly of the red carrot variety TouXinHong4 (TXH4), generated using a combination of PacBio high-fidelity (HiFi) sequencing, high-throughput chromosome conformation capture (Hi-C) data, and Oxford Nanopore Technology (ONT) ultra-long reads. The assembled carrot genome was subjected to gene prediction and comparative genomic analyses. TXH4, a red carrot landrace widely grown in western China, is recognized for its excellent storage properties, cold tolerance, and appealing flavor. Carotenoid analysis revealed that decreased levels of both α-carotene and β-carotene led to lycopene accumulation in TXH4. Further transcriptomic analysis of *DcLCYE* and *DcLCYB1* indicated that these genes were involved in lycopene metabolism. This research highlights the accuracy of the TXH4 genome, enhancing our understanding of the genomic diversity within carrot germplasm and supporting more efficient genome-assisted breeding strategies for crop improvement.

## Results

### Genome assembly and evaluation

**Figure 1:**
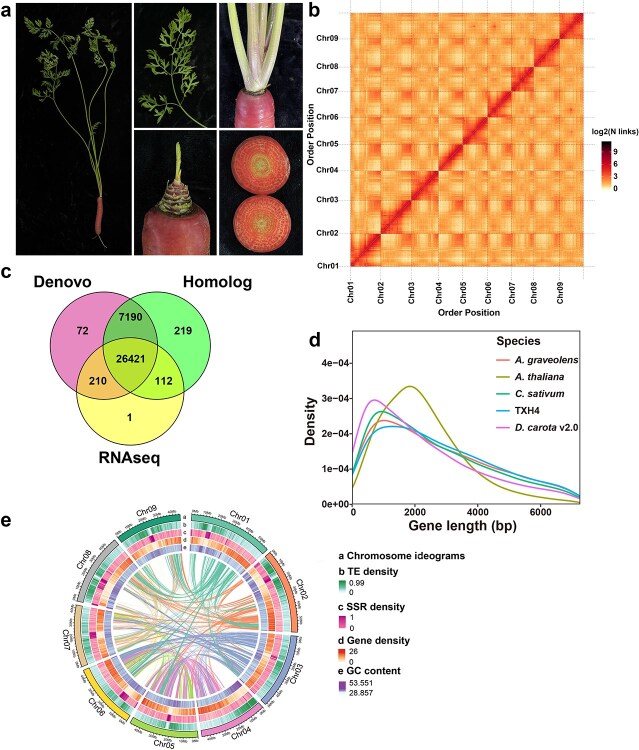
Characteristics of TXH4 genome assembly and annotation. (a) Images showing the entire plant, leaf, stem, petiole, and root of TXH4. (b) The Hi-C interaction heatmap illustrating the *pseudo*-chromosome assembly of TXH4, with interaction strength calculated at a resolution of 100 kb per bin. (c) The number of coding genes identified through *de novo* prediction, homology-based comparison, and transcriptome-based prediction. (d) Comparative analysis of gene lengths across different species, such as *A. thaliana*, *A. graveolens*, *C. sativum*, TXH4, and *D. carota* v2.0. (e) Features of the TXH4 genome. The visualization displays the nine chromosomes (Chr01–Chr09) of the TXH4 genome in distinct colors, highlighting the syntenic relationships between chromosomes. Physical coordinates are marked at 20 Mb intervals along each chromosome. Four color-coded layers correspond to transposable element (TE) density, simple sequence repeat (SSR) density, gene density, and GC content, with increasing color intensity indicating higher density values.

The red carrot TXH4 is a high-generation inbred line derived from the landrace TouXinHong and is characterized by its red, cylindrical fleshy root (Fig. 1a). The good storage resistance and cold tolerance of TXH4 make it well-suited to the climate of western China, leading to its widespread cultivation in this area.

**Figure 2:**
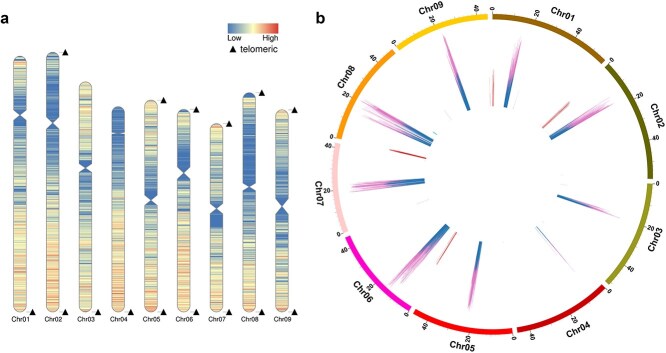
Localization of telomeres and centromeres in the TXH4 genome. (a) Telomere positions on chromosomes, indicated by triangles. The colors on the chromosomes represent gene density, and the indentations indicate the positions of the centromeres. (b) Centromere positions on chromosomes, marked with blue lines.

To achieve a high-quality genome assembly of TXH4, we employed a multi-sequencing approach. Initially, we generated approximately 60× coverage of Illumina paired-end short reads (150 bp) to evaluate the general characteristics of the genome. This analysis revealed that the genome size of TXH4 was around 410.54 Mb, with heterozygosity and repeat sequence percentages of 0.25% and 44.07%, respectively (Supplementary Fig. S1). Next, we performed deep sequencing using PacBio HiFi reads, ONT ultra-long reads, and high throughput chromatin capture (Hi-C) sequencing. This process integrated 36.51, 20.55, and 57.63 Gb of HiFi reads, ONT ultra-long reads, and Hi-C data, respectively (~88×, ~49×, and ~ 138× coverage, respectively). Firstly, we generated a preliminary assembly of the carrot genome. Evaluation of this preliminary assembly indicated a contig N50 of 47.30 Mb, demonstrating good integrity and sequencing uniformity (Supplementary Table S1; Supplementary Fig. S2). Then, we employed the HiC-Pro program to construct chromosomal interaction maps. Using these interaction signals, sequences of a total length of 449.42 Mb could be associated to nine chromosomes, and the final TXH4 genome assembly is gap-free (Fig. 1b; Supplementary Table S1). Next, we evaluated the integrity of the genomic assembly using CEGMA and BUSCO analyses, resulting in successful matching for 98.69% and 98.70% of the conserved genes, respectively (Supplementary Table S2). These findings confirmed the high quality of the TXH4 genome assembly.

### Repeat sequence and gene structure

Most of the repetitive sequences in the carrot genome were tandem repeats and transposable elements (TEs). We reconstructed a carrot-specific repeat sequence database by combining ab initio prediction with known repeat databases. After removing redundancy, we identified 172.56 Mb of TE sequences and 47.44 Mb of tandem repeat sequences, accounting for 38.15% (Supplementary Table S3) and 10.49% (Supplementary Table S4) of the carrot genome, respectively. The primary components of TE sequences were retroelements, including LTR-Copia and LTR-Gypsy, which together accounted for 56.09% of the total TE sequences and 21.41% of the entire genome (Supplementary Table S3). Satellites are the primary component of tandem repeat sequences, making up 63.05% of all repeat sequences and 6.61% of the whole genome (Supplementary Table S4). However, microsatellites and minisatellites are more widely distributed throughout the genome than satellite repeats. Overall, tandem repeat sequences provide valuable information for subsequent molecular marker development.

We then used the repeat-masked genome to annotate gene structure by combining *de novo* prediction, homology comparison (with genomes of *Arabidopsis thaliana*, *Coriandrum sativum*, *Daucus carota* v2.0, and *Apium graveolens*), and transcriptome-based prediction. After integrating results and removing redundancies with the EVidenceModeler pipeline, we predicted 34 225 protein-encoding genes in the carrot genome (Supplementary Table S5). We then counted the number of coding genes identified by the three prediction approaches, discovering that the majority of genes were supported by at least two methods. A total of 26 421 genes (77.20%) were supported by all three prediction approaches (Fig. 1c). The predicted genes accounted for approximately 31.2% (~137 Mb) of the entire genome. On average, each gene had a length of 4014.38 bp, with the average lengths of coding sequences (CDSs) and introns being 1256.23 and 2335.40 bp, respectively, and each gene containing an average of 4.3 exons (Supplementary Table S6). We also examined the length distribution of key gene structure components (genes, CDSs, mRNAs, and introns) in the T2T genome of TXH4 by comparing it with those of *A. thaliana*, *C. sativum*, *D. carota* v2.0, and *A. graveolens*. The results indicated that the frequency distribution of gene structure lengths in the TXH4 genome was generally consistent with those in the other genomes, except for some differences in the intron length distribution in the *A. thaliana* genome (Supplementary Fig. S3). Additionally, compared to the *D. carota* v2.0 genome, the TXH4 T2T genome was significantly optimized in terms of gene structure (Fig. 1d; Supplementary Fig. S3). After annotating the genes and repeat sequences, we generated a circos map to illustrate various genome features, including collinearity within the carrot genome, TE density (ranging from 0 to 0.99), simple sequence repeat (SSR) density (ranging from 0 to1), gene density (ranging from 0 to 26) and GC content (ranging from 28.89% to 53.55%) (Fig. 1e).

### Pseudogenes, non-coding RNAs, and gene function annotation

To annotate the predicted genes, we used multiple protein databases, including the National Center for Biotechnology Information (NCBI) Non-Redundant (NR), eggNOG, Gene Ontology (GO), Kyoto Encyclopedia of Genes and Genomes (KEGG), TrEMBL, EuKaryotic Orthologous Groups (KOG), SWISS-PROT, and Pfam. As a result, 34 016 genes (99.39%) were successfully matched to these databases, and we obtained at least one annotation with each database (Supplementary Table S7). The sequences of pseudogenes are similar to those of functional genes, but the former eventually lost their functions due to insertions, deletions, or other mutations. To predict pseudogenes, we used GenBlastA to identify homologous gene sequences (potential genes) within the real-gene-masked genome and screened these sequences for frameshift mutations and premature stop codons. We predicted a total of 137 pseudogenes (Supplementary Table S8), with an average length of 3078 bp. Additionally, we analyzed non-coding RNAs, such as microRNAs (miRNAs), rRNAs, and tRNAs, using various strategies tailored to each type’s structural features. Overall, we annotated 633 tRNAs, 3222 rRNAs, and 130 miRNAs (Supplementary Table S9).

### Identification of telomeres and centromeres

Telomeres, composed of short tandemly arranged minisatellites, are a conserved feature of plant genome sequences. We detected 15 telomeres at the ends of the nine chromosomes using telomere repetitive sequences (CCCATTT at the 5′ end and TTTAGGG at the 3′ end) as queries. Chr01, Chr03, and Chr04 each harbored a single terminal with telomere structures, while the other six chromosomes were assembled from one telomere to the next (Fig. 2a; Table 1).

**Table 1:** Telomeres and centromeres in the TXH4 genome.

**Chr**	**Telomeres**							**Centromeres**		
	**Chr length (bp)**	**Left start (bp)**	**Left end (bp)**	**Left length (bp)**	**Right start (bp)**	**Right end (bp)**	**Right length (bp)**	**Start (bp)**	**End (bp)**	**Length (bp)**
Chr01	57 104 046				57 103 619	57 104 046	428	12 028 646	14 313 910	2 285 264
Chr02	57 990 488	1	11 510	11 510	57 987 073	57 990 488	3416	14 450 838	17 160 123	2 709 285
Chr03	51 360 713				51 359 337	51 360 713	1377	18 255 310	20 201 450	1 946 140
Chr04	45 846 886				45 846 028	45 846 886	859	5 760 381	6 143 452	383 071
Chr05	47 295 712	1	16 628	16 628	47 291 620	47 295 704	4085	21 203 877	23 499 016	2 295 139
Chr06	45 237 041	1	35 554	35 554	45 227 622	45 237 041	9420	12 875 268	15 548 224	2 672 956
Chr07	42 057 369	1	1098	1098	42 055 043	42 057 369	2327	17 843 072	20 130 423	2 287 351
Chr08	48 947 466	22	1343	1322	48 947 302	48 947 466	165	20 182 346	21 868 776	1 686 430
Chr09	45 119 215	1	449	449	45 113 976	45 119 215	5240	19 814 852	23 295 694	3 480 842

Centromeres are regions on chromosomes during cell division where spindle microtubules attach. They are characterized by a high concentration of DNA repeats, typically 100 to 1000 bp in length. Identifying centromere regions provides valuable insights into genome architecture, karyotype evolution, and the recombination and segregation processes during inter-species carrot hybridization. The T2T assemblies enabled a detailed analysis of these regions. Using the centromics pipeline, we identified tandem repeats along the chromosomes and clustered them to reduce sequence redundancy. This approach allowed us to identify and annotate the centromere regions on each chromosome (Fig. 2b; Table 1). The length of centromeres varied significantly among the chromosomes, ranging from 0.38 Mb on Chr04 to 3.48 Mb on Chr09 (Table 1). Meanwhile, we observed that centromeres on chromosomes frequently coincided with regions of higher SSR density. This finding indicated that centromeric regions were enriched with repetitive sequences.

### Comparative genomic analyses

**Figure 3:**
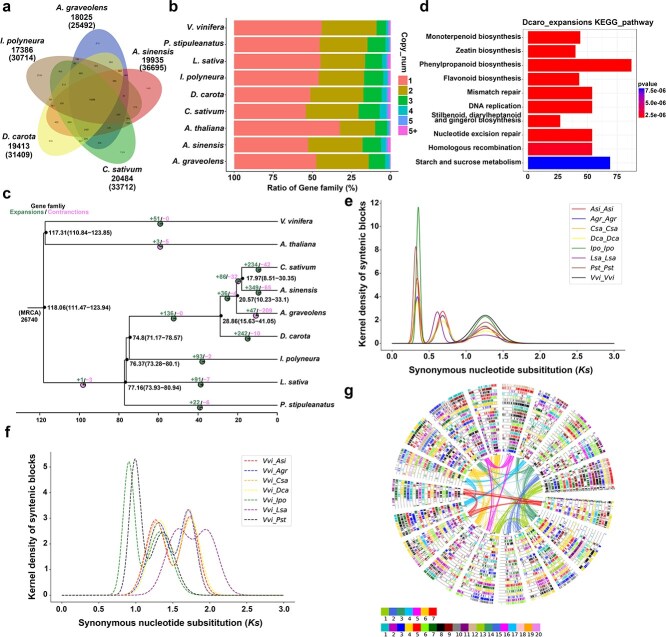
Comparative genomics and evolutionary analysis across nine species. (a) Gene family clustering among carrot (*D*. *carota*) and its relatives, including *A. sinensis*, *C. sativum*, and *A. graveolens*, with *I. polyneura* as the outgroup. (b) Distribution of gene family copy numbers across species. (c) Phylogenetic tree showing divergence timelines, gene family expansions, and contractions among the nine species. (d) KEGG enrichment analysis of 242 expanded gene families in carrots. (e) Synonymous nucleotide substitution rate (KS) analysis among Apiaceae species. (f) Genome-wide collinearity analysis between species using *V. vinifera* as a reference. The sub-genomes are delineated based on the number of whole-genome duplication (WGD) events. (g) Circular diagram illustrating the collinearity of homologous regions between other species and *V. vinifera*. The inner ring represents *V. vinifera* chromosomes, with collinear genes mapped for each species. The curve of the inner ring was made up of 19 corresponding grape chromosomes.

To elucidate the connection between the biological evolution of species and their specific biological functions, we compared the protein-encoding genes from nine different species: *A. graveolens*, *Angelica sinensis*, *A. thaliana*, *C. sativum*, *D. carota*, *Ilex polyneura*, *Lactuca sativa*, *Panax stipuleanatus*, and *Vitis vinifera*. Through sequence similarity analysis, we identified 31 409 genes in the carrot grouped into 19 413 gene families, including 569 gene families unique to the carrot. Additionally, 4609 gene families were found to be conserved across all nine species (Fig. 3a; Supplementary Table S10). For a better insight into the clustering of gene families across species, we conducted a comparative analysis focusing on three carrot relatives (*A. sinensis*, *C. sativum*, and *A. graveolens*) and one outgroup species (*I. polyneura*). This analysis identified 10 586 shared gene families among these species, including 836 unique to the carrot (Fig. 3a; Supplementary Table S10). To explore the genome structure and evolutionary history of different species, we examined changes in the gene family numbers among them. Our findings showed that in *Arabidopsis*, the majority of gene families existed as single copies (~70%), consistent with the characteristics of early evolutionary species (Fig. 3b). In contrast, Apiaceae plants, which evolved later, harbored a significantly higher proportion of multi-copy gene families (~50%).

Next, to investigate the genetic relationships and divergence times between carrots and other species, we built a phylogenetic tree using 1363 conserved single-copy genes (Fig. 3c). This analysis shed light on the evolutionary history of the nine species. As expected, the four Apiaceae species (*C. sativum*, *A. sinensis*, *A. graveolens*, and *D. carota*) formed a single clade, diverging from *I. polyneura* approximately 74.8 million years ago (Mya). The divergence between *C. sativum* and *A. sinensis* occurred more recently, around 17.97 Mya. During the evolution of Apiaceae from lettuce-related species, we identified 242 gene families that had expanded and ten that had contracted in carrots (Fig. 3c). KEGG enrichment analysis showed significant enrichment (*P* < 1e−5) of the pathways involved in monoterpenoid, zeatin, phenylpropanoid, and flavonoid biosynthesis (Fig. 3d), indicating that these gene families contribute to shaping the unique nutritional properties, flavors, and tastes of carrots.

### Genomic collinearity and evolution analysis

We explored the genome evolution of Apiales by calculating the synonymous nucleotide substitution rate (KS) (Fig. 3e). Species within the family Apioideae, such as *A. graveolens*, *A. sinensis*, *C. sativum*, and *D. carota*, experienced three shared duplication events, one of which was a triplication event (γ). In addition, we also probed species differentiation based on the peak KS values between species. Given the significant differences in the rate of evolution between different species, the assessment of evolutionary time may be affected. Therefore, we used the evolutionary events shared among species of the order Apiales as the correction benchmark to infer a relatively accurate evolutionary time of species. Based on the doubling event experienced by plants, the KS values of the homologous genes of all Apiales plants were corrected, and the KS peaks were fitted. Based on this finding, the two diploidization events were corrected based on the whole genome duplication (WGD) events common to *A. graveolens*, *A. sinensis*, *C. sativum*, and *D. carota,* and the time nodes of each key evolutionary time were inferred. After three rounds of correction, we aligned the KS peaks of the doubling events common to the family at 0.33, 0.68, and 1.25 Mya.

We used the ‘-icl’ program in the WGDI software to assess the genome collinearity and the ‘-ci’ program to demonstrate the collinearity between species. Using *V. vinifera* as a reference, a global alignment of homologous regions across the genomes of these species was performed. Since all seven plant species underwent genome-wide doubling events, their genomes were further divided into sub-genomes (Fig. 3f). Since *V. vinifera* only experienced a single ancient whole genome triplication (WGT) event, its genome was used as a reference. Each of the *A. graveolens*, *A. sinensis*, *C. sativum*, and *D. carota* genomes was further divided into four sub-genomes, as each of them experienced two additional WGD events after diverging from *V. vinifera*. Similarly, the *L. sativa* genome was split into three sub-genomes due to an additional WGT event following its divergence from *V. vinifera*. Each of the *I. polyneura* and *P. stipuleanatus* genomes was further divided into two sub-genomes, reflecting a single additional WGD event following their divergence from *V. vinifera*. Collinear genes between the sub-genomes of each species and *V. vinifera* are illustrated in Fig. 3g.

**Figure 4:**
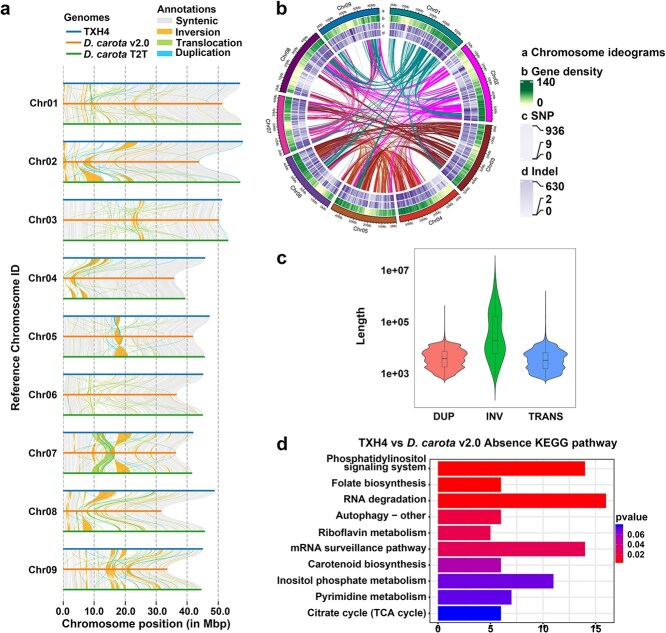
Comparative genomic analysis and variation assessment in carrot genomes. (a) Alignment of collinear regions and detection of structural rearrangements, including inversions, translocations, and duplications, among the TXH4 genome, *D. carota* T2T (Kurodagosun), and *D. carota* v2.0 (Nantes-type doubled-haploid carrot DH1). (b) Distribution patterns of SNPs and InDels in *D. carota* T2T and *D. carota* v2.0 genomes compared to the TXH4 reference genome. (c) Structural variations identified in the TXH4 genome. (d) KEGG pathway enrichment analysis of presence variations uniquely identified in the TXH4 genome.

### Genomic diversity among carrot varieties

To gain deeper insights into the genomic differences and diversity among carrot varieties, we utilized the TXH4 genome assembled in this study as a reference. We compared the TXH4 genome with two previously assembled genomes, *D. carota* T2T (Kurodagosun) [21], and *D. carota* v2.0 (Nantes-type doubled-haploid carrot DH1) [22], to identify collinear regions, structural rearrangements (including inversions (INV), translocations (TRANS), and duplications (DUP)), and local variations (such as gene density, single nucleotide polymorphisms (SNPs), and insertion/deletions (InDels)) relative to the TXH4 genome (Fig. 4a). Our analysis revealed 2 466 422 and 2 037 986 SNPs (Fig. 4b; Supplementary Table S11) and 500 579 and 474 704 InDels (Fig. 4b; Supplementary Table S12) in *D. carota* T2T and *D. carota* v2.0, respectively. Most of these variations were located in intergenic and intronic regions. A total of 17 875 structural variations (SVs) were identified in DcT2T, comprising 6290 TRANS, 83 INV, and 11 502 DUP. In addition, 17 433 SVs were detected in DcRef, including 5779 TRANS, 100 INV, and 11 554 DUP (Supplementary Table S13). Among these, INV exhibited a more extensive length distribution compared to TRANS and DUP (Fig. 4c). We further investigated variations specific to the *D. carota* T2T and *D. carota* v2.0 genomes, identifying 3401 and 4048 presence variations unique to *D. carota* T2T and *D. carota* v2.0, respectively (Supplementary Table S14). KEGG pathway enrichment analysis of the 4048 presence variations unique to *D. carota* v2.0 showed significant associations with the phosphatidylinositol signaling, folate biosynthesis, and carotenoid biosynthesis pathways (*P* < 0.05), (Fig. 4d). The accumulation of unique variations in the carotenoid biosynthesis pathway suggested that the carotenoid accumulation pattern in TXH4 was distinct from that of orange carrots, such as Nantes and ‘Kurodagosun’.

**Figure 5:**
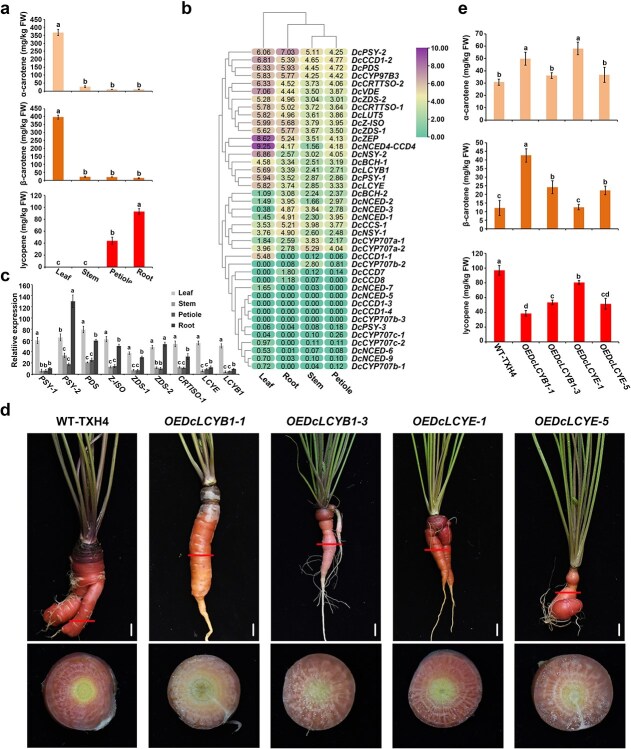
Carotenoid biosynthesis and the effects of *DcLCYB1* and *DcLCYE* overexpression in TXH4. (a) α-carotene, β-carotene, and lycopene contents in various TXH4 tissues, including leaf, stem, petiole, and root. All data are presented as mean values ± standard deviations (SD) based on three biological replicates. (b) Heatmaps displaying relative fold changes in the expression of structural genes involved in the carotenoid biosynthesis pathway across all TXH4 tissues. (c) Expression profiles of structural genes involved in carotenoid biosynthesis in leaf, stem, petiole, and root of TXH4. Each measurement was performed in triplicate, with data represented as mean ± SD. (d) Morphological phenotypes of plants overexpressing *DcLCYB1* and *DcLCYE*. (e) α-carotene, β-carotene, and lycopene levels in wild-type (WT) TXH4 and *DcLCYB1*- and *DcLCYE*-overexpressing roots. All data are reported as mean values ± SD from three biological replicates. Significant differences (*P* < 0.05) were assessed using ANOVA followed by Tukey’s HSD test and are marked by distinct letters.

### Carotenoid biosynthesis in TXH4 tissues

The red coloration of the TXH4 root distinguishes it as a unique carrot variety from western China. Previous studies have suggested that this red flesh results from lycopene accumulation. To investigate this, we measured carotenoid contents in various TXH4 tissues using ultra-performance liquid chromatography (UPLC) (Fig. 5a, Supplementary Fig. S4, and Table S15). The results revealed that lycopene content in TXH4 roots reached 93.1 mg/kg fresh weight (FW), whereas lycopene content in the leaves was only 0.2 mg/kg FW, indicating that lycopene levels in the root were 1965 times higher than those in the leaves. In contrast, the contents of α-carotene and β-carotene in the leaves were 367.6 and 396.6 mg/kg FW, respectively, while those in the roots were only 10.1 and 13.7 mg/kg FW, accounting for just 2.7% and 3.5% of the levels in the leaves. These findings suggested that TXH4 roots accumulate large amounts of lycopene, and that carotenoid biosynthesis is regulated differently in roots and leaves.

**Figure 6:**
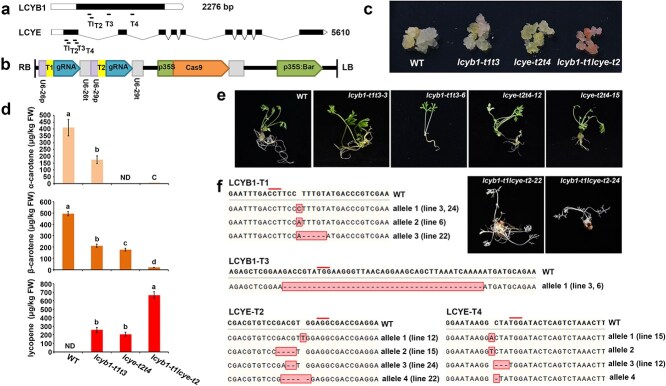
Knockout of *DcLCYB1* and *DcLCYE* in orange carrot ‘Kurodagosun’. (a) Schematic diagram of the *DcLCYB1* and *DcLCYE* genes, highlighting the Cas9 gene-editing target sites indicated by lines. (b) Physical map and structure of the *DcLCYB1* and *DcLCYE* gene editing Cas9 vector. U6-26p and U6–29p are two AtU6 gene promoters; U6-26 t and U6-29t are corresponding AtU6 gene terminators; p35S, CaMV*35S* promoter; Bar, bialaphos resistance gene. (c) Phenotypes of the callus tissue following the knockout of *DcLCYB1* and *DcLCYE*. (d) Quantification of α-carotene, β-carotene, and lycopene levels in the edited callus tissues. All data are based on three biological replicates and presented as mean values ± SD. Statistical significance (*P* < 0.05) was assessed using ANOVA followed by Tukey’s HSD test and is indicated by distinct letters. ND: not detected. (e) The seedling phenotypes resulting from the knockout of *DcLCYB1* and *DcLCYE*. (f) Identification of gene-editing mutants for *DcLCYB1* and *DcLCYE*, with PAM sequences marked in lines and mutated sequences highlighted within boxes.

### Expression pattern of carotenoid structural genes in TXH4

To predict gene structures and their genomic locations, transcriptome analyses were conducted using various tissues of TXH4, including leaves, stems, petioles, and roots. In parallel, a comparative transcriptional analysis was performed to investigate the expression patterns of key structural genes involved in the carotenoid biosynthesis pathway (Fig. 5b). To validate the transcriptome data, quantitative real-time PCR (qRT-PCR) was conducted (Fig. 5c). The results showed that all examined genes exhibited relatively high expression levels in the leaves. In contrast, in the roots, with the exception of *DcPSY1*, the expression levels of the genes related to lycopene biosynthesis positively correlated with the lycopene content. *DcPSY1* and *DcPSY2* displayed similar expression levels in the leaves; however, in the roots, *DcPSY1* was significantly downregulated while *DcPSY2* was strongly upregulated (Fig. 5b and c). *DcLCYE* and *DcLCYB1*, which catalyze the cyclization of lycopene into α-carotene and β-carotene, were significantly downregulated in the roots compared to the leaves, and their expression levels negatively correlated with lycopene accumulation. These findings suggested that the downregulation of *DcLCYE* and *DcLCYB1* is a critical factor contributing to the high lycopene accumulation in TXH4 roots.

### Effects of overexpression of *DcLCYB1* and *DcLCYE* on lycopene levels in carrot

We overexpressed *DcLCYB1* and *DcLCYE* in TXH4 to elucidate their roles in lycopene accumulation. The *OEDcLCYB1-1* and *OEDcLCYB1-3* lines exhibited the highest *DcLCYB1* expression, while *OEDcLCYE-1* and *OEDcLCYE-5* exhibited the highest *DcLCYE* expression (Supplementary Fig. S5). Compared with the red fleshy root of TXH4, the roots of the *DcLCYB1* and *DcLCYE* overexpression lines showed a lighter red color, with the root tip of *OEDcLCYB1-1* showing a noticeable shift to orange. Additionally, the petiole color of TXH4 was dark red, but the overexpression of *DcLCYB1* and *DcLCYE* resulted in a lighter petiole color (Fig. 5d). Carotenoid analysis demonstrated that the overexpression of *DcLCYB1* or *DcLCYE* in TXH4 led to a significant reduction in lycopene levels. Meanwhile, *OEDcLCYB1-1*, *OEDcLCYB1-3*, and *OEDcLCYE-5* exhibited increased accumulation of both α-carotene and β-carotene. In contrast, *OEDcLCYE-1* specifically showed an elevated α-carotene content (Fig. 5e, Supplementary Fig. S6, and Table S16). These results suggested that enhanced expression of *DcLCYB1* or *DcLCYE* in TXH4 roots promoted the conversion of lycopene into α-carotene and β-carotene.

### Effects of knockout of *DcLCYB1*and *DcLCYE* on lycopene accumulation in carrot

To further investigate the roles of *DcLCYB1* and *DcLCYE* in carotenoid biosynthesis, we utilized a CRISPR/Cas9-based system to introduce targeted mutations into these genes. Four target sites were designed for each gene (Fig. 6a), cloned into a gene-editing vector (Fig. 6b), and subsequently used to transform the orange carrot variety ‘Kurodagosun’. In wild-type callus tissue, a light yellow coloration was observed. *DcLCYB1* knockout resulted in the callus tissue with a slightly red color, whereas *DcLCYE* knockout produced no noticeable color change. However, when both *DcLCYB1* and *DcLCYE* were simultaneously knocked out, the callus tissue exhibited a pronounced red coloration (Fig. 6c).

Carotenoid measurements revealed that the carotenoid content in the callus tissue was significantly lower than that in whole carrot plants. In the callus tissue of *DcLCYB1* knockout lines, both α-carotene and β-carotene levels decreased significantly, while lycopene levels increased markedly, consistent with the observed slight red coloration in the callus tissue. In the callus tissue of *DcLCYE* knockout lines, α-carotene became nearly undetectable, β-carotene levels significantly decreased, and lycopene levels increased sharply, indicating that lycopene is metabolized exclusively through the β-carotene branch. When both *DcLCYB1* and *DcLCYE* were knocked out, α-carotene and β-carotene accumulation was minimal, whereas lycopene accumulated extensively, leading to a distinct red coloration in the callus tissue (Fig. 6d, Supplementary Fig. S7, and Table S17).

Single mutants of *DcLCYB1* and *DcLCYE* were able to grow normally, but double mutants exhibited albinism and were unable to grow normally (Fig. 6e and f). Although single mutants exhibited hindered carotenoid accumulation and reduced lycopene accumulation, the effects were more pronounced in the double mutants. These findings showed that *DcLCYE* and *DcLCYB1* share some functional redundancy in converting lycopene into α-carotene and β-carotene. Consequently, the disruption of both genes resulted in significantly higher lycopene accumulation than single mutants.

## Discussion

Carrot is an important crop in the Apiaceae family and is cultivated worldwide. Domesticated carrots are believed to have originated in Central Asia and are traditionally classified into two types, eastern and western. The western orange carrot is thought to have been selected from yellow domesticated varieties [1, 5]. In contrast, the Chinese orange carrot might have evolved from the Chinese red carrot, following a distinct domestication pathway separate from that of the western orange carrot [9]. Given the diverse evolutionary history of carrots, high-quality genomes from various germplasms are crucial for advancing molecular breeding and ensuring successful breeding programs. T2T assemblies of two orange carrots, ‘Nantes’ [23] and ‘Kurodagosun’ [21], with genome sizes of 451.04 and 430.4 Mb, respectively, have already been reported. In this study, we assembled a high-quality T2T genome for the red carrot variety TXH4, a highly inbred line with a total genome size of 449.42 Mb. Our results provided valuable insights into the red carrot genome and enriched the genomic resources available for carrot breeding, enhancing our understanding of genetic variation in the breeding pool.

Carrots are considered a model system for studying carotenoid biosynthesis [28]. Genes encoding the enzymes involved in the carotenoid biosynthesis pathway directly impact carotene production. LCYB and LCYE are key enzymes involved in converting lycopene into other carotenoids. In carrots, *LCYE* exists as a single-copy gene, while *LCYB* has two copies. In the current study, *DcLCYB2* expression was undetectable in all carrot tissues. Previous studies on the red carrot variety ‘Benhongjinshi’ revealed that reduced *DcLCYE* expression led to increased lycopene levels [32]. In this inbred line, α-carotene levels decreased, whereas β-carotene levels remained comparable to or were slightly higher than those in orange carrots. In contrast, TXH4 exhibited decreased levels of both α-carotene and β-carotene. This finding suggested that although lycopene accumulation caused red coloration in the fleshy roots of both TXH4 and ‘Benhongjinshi’, the mechanisms underlying lycopene accumulation in these two inbred lines were distinct. *DcLCYB1* overexpression in TXH4 roots promoted the conversion of lycopene into both α-carotene and β-carotene, whereas *DcLCYE* overexpression specifically promoted the transformation of lycopene into α-carotene. On the other hand, the expressions of both *DcLCYB1* and *DcLCYE* were nearly absent in TXH4 roots, resulting in minimal accumulation of both α-carotene and β-carotene. High lycopene accumulation in TXH4 can be attributed to the decreased levels of both α-carotene and β-carotene, primarily due to the downregulation of *DcLCYB1* and *DcLCYE*.

LCYB was the first enzyme in both branches, converting lycopene into α-carotene and β-carotene, and in theory, its functional disruption significantly impacted carotenoid synthesis. However, CRISPR/Cas9 knockout experiments revealed that knocking out both *DcLCYE* and *DcLCYB1* caused the callus tissue to accumulate a strong red color, while the plants displayed a pronounced albino phenotype. This phenotype was much more prominent in the double mutants than in mutants with only *DcLCYB1* knocked out, which caused a slight red coloration in the callus with normal leaf development. These findings suggested that in addition to DcLCYB, other enzymes might work in synergy with DcLCYE to catalyze the conversion of lycopene into α-carotene. While the potential involvement of DcLCYB2 remains unclear and requires further investigation, it was evident that DcLCYB2 does not contribute to carotenoid synthesis in carrot leaves.

It is worth noting that carotenoid contents often differ between naturally grown and tissue-cultured carrots. A comparison between Fig. 5a and e revealed a marked difference in the α-carotene levels but relatively consistent β-carotene and lycopene contents. This variation might be attributed to the differences in growth conditions. In Fig. 5a, TXH4 was grown under natural conditions, whereas in Fig. 5e, WT-TXH4 plants were subjected to dedifferentiation and redifferentiation via tissue culture to control for potential artifacts and ensure a fair comparison with *DcLCYB1* and *DcLCYE* overexpression lines. In addition to the variations in carotenoid levels, significant changes in root morphology, including enhanced branching and twisting, were observed in both wild-type and transgenic lines in Fig. 5e. These variations in carotenoid content and root morphology were consistent with previous studies [32] and are likely caused by stress or hormonal effects associated with the tissue culture process.

## Conclusion

This study presents a high-quality, telomere-to-telomere genome assembly of the Chinese red carrot TXH4, providing valuable insights into the genetic basis of lycopene accumulation. Our findings revealed that the downregulation of *DcLCYB1* and *DcLCYE* crucially contribute to the red root phenotype of TXH4. Functional validation through gene overexpression and knockout experiments further confirmed the roles of these genes in lycopene metabolism. Our results not only expand the genomic resources for carrot breeding but also enhances our understanding of carotenoid biosynthesis, offering valuable implications for crop improvement and biofortification.

## Materials and methods

### Plant materials

The leaf, stem, petiole, and root of the red carrot inbred line TXH4 were used for genomic and transcriptomic analyses as well as carotenoid content assessment. The orange carrot inbred variety ‘Kurodagosun’ was utilized as the material for transformation. TXH4 and ‘Kurodagosun’ were developed through forced selfing over multiple generations. Both TXH4 and ‘Kurodagosun’ were grown under natural conditions in the experimental field of the Vegetable Research Center at the Beijing Academy of Agriculture and Forestry Sciences. DNA samples for genome analysis were extracted from the leaves of a single healthy carrot plant at 90 days.

### Genome assembly

A 150-bp short-read library was prepared for Illumina sequencing on the NovaSeq platform to obtain clean reads. The quality-filtered reads were then used to estimate the genome size, which was determined to be 410.54 Mb, with a heterozygosity rate of 0.25%.

A long-read library for PacBio HiFi sequencing was prepared from fragmented genomic DNA (15 kb) according to the manufacturer’s protocol. The sequencing was carried out on the PacBio Sequel II platform, and the resulting subreads were cleaned by removing the low-quality reads and sequence adapters. CCS reads were then generated for subsequent analysis. For ONT ultra-long sequencing, the DNA samples were sequenced using the Nanopore sequencing platform [33]. Low-quality reads were discarded, and fragments shorter than 70 kb were removed. The remaining reads with mean quality scores above 90% were then used for genome assembly. For Hi-C sequencing, raw data was obtained using the Illumina high-flow sequencing platform [34], which is based on sequencing-by-synthesis (SBS) technology. The raw Hi-C data were filtered to remove low-quality reads, and clean read pairs were then aligned to the initially assembled TXH4 genome using the BWA [35].

Genome contig sequences were generated by assembling high-accuracy CCS data, Hi-C data, and ultra-long ONT reads using the hifiasm (v0.19.1) software. The best assembly was selected and subjected to blast against the nt, mitochondrial, and plastid databases to remove contamination sequences. Then, the Lachesis software [34] was used to scaffold the contigs into a chromosome-level assembly using the Hi-C data. Gaps in the chromosome-level genome were filled using the quarTeT software (https://github.com/aaranyue/quarTeT) based on other contig-level genome versions, resulting in the final gap-free T2T genome.

The completeness of the assembled genome was evaluated using BUSCO v5.2.2 [36] and CEGMA v2.5 [37]. Illumina short reads were aligned to the genome to confirm the quality of the contig assembly. Additionally, the Hi-C [38] data were applied to anchor and scaffold the contigs, with Lachesis employed for clustering, ordering, and orienting the contigs.

All sequencing was performed by Biomarker Technologies Co., Ltd (Beijing, China).

### Identification of centromeres

Following the principle that centromeric regions consist of highly abundant tandem repeats, we identified these regions in the genome using the Centromics (https://github.com/ShuaiNIEgithub/Centromics) with the following parameters: -min_ratio 0.03, −k 50 000, and -p 500. First, CCS data were extracted and analyzed using Tandem Repeats Finder (TRF) to identify tandem repeat sequences. These sequences were then clustered to determine potential repeat units. The genome was subsequently aligned to the TRF sequences to locate corresponding centromeric regions.

### Annotation of repetitive sequences and protein-coding genes

TEs and tandem repeats were annotated through integrated homology-based and *de novo* approaches. A custom TE library was built with RepeatModeler, integrating RECON and RepeatScout. Full-length long terminal repeats (fl-LTRs) were identified with LTRharvest [39] and LTR_FINDER [40]. High-quality fl-LTRs were further refined using LTR_retriever [41], and the final TE library was created by merging species-specific sequences with the Dfam database. TE sequences were classified using RepeatMasker [42]. For tandem repeat identification, Tandem Repeats Finder [43] and MISA [44] were employed.

Protein-coding genes were annotated using a combination of *de novo* prediction (Augustus [45] and SNAP [46]), homology search (GeMoMa [47]), and transcript-based assembly (Hisat [48] and Stringtie [49]). Gene refinement was performed using GeneMarkS-T [50] and Trinity-based [51] full-length transcripts. The gene models from these approaches were combined using EVM [52] and updated with PASA [53], resulting in 34 225 protein-coding genes with an average length of 4014.38 bp, covering 31.2% of the genome.

### Functional annotation of protein-coding genes

Gene functions were annotated by aligning the predicted proteins to several databases, including NCBI NR (ftp://ftp.ncbi.nlm.nih.gov/blast/db), EggNOG (http://eggnog5.embl.de/download/eggnog_5.0/), EuKaryotic Orthologous Groups (KOG; http://www.ncbi.nlm.nih.gov/KOG/), TrEMBL (http://www.uniprot.org/), Swiss-Prot (http://ftp.ebi.ac.uk/pub/databases/swissprot), and Kyoto Encyclopedia of Genes and Genomes (KEGG; http://www.genome.jp/kegg) using Diamond [54] (*E*-value ≤1E−3). Protein domains were identified with InterProScan and PFAM (v33.1, http://pfam.xfam.org). Gene ontology (GO, http://www.geneontology.org/) terms were assigned from TrEMBL, InterPro, and EggNOG. As a result, 34 016 (99.39%) protein-coding genes were successfully annotated with known genes, conserved domains, and GO terms.

### Annotation of non-coding RNA genes and pseudogene prediction

The tRNAs were detected using tRNA scan-SE (v1.3.1) [55], and rRNAs were annotated with Barrnap (v0.9) [56], both using default settings. Small nucleolar RNAs (snoRNAs), micro-RNAs (miRNAs), and small nuclear RNAs (snRNAs) were identified using Infernal (v1.1) [57] by querying the Rfam database (http://rfam.xfam.org/). Pseudogenes were predicted with GenBlastA (v1.0.4) [58] by scanning the masked genome, followed by the identification of frameshift mutations and premature stop codons with GeneWise (v2.4.1) [59].

### Gene family identification and phylogenetic analysis

Protein sequences derived from the longest transcripts of each gene in TXH4 and related species were classified into gene families using OrthoFinder (v2.4.0) [60]. Pfam V33.1 [61] was used to annotate these gene families. This analysis revealed 19 413 gene families, including 569 single-copy orthologues.

Phylogenetic relationships between carrot and related species were reconstructed using 1363 single-copy orthologous genes. Protein sequences were aligned using mafft (v7.205) [62], and corresponding coding sequence (CDS) alignments were concatenated based on the protein alignment. The phylogenetic tree was built using IQ-TREE [63], revealing relationships consistent with those elucidated by previous studies.

### Gene family expansion and contraction analysis

Gene family expansion and contraction in carrot TXH4 were investigated using CAFE (v4.2) [64] with a random birth-death model based on phylogenetic relationships and divergence times. Gene families exhibiting a conditional *P*-value ≤0.05 were identified as significantly expanded or contracted. A hypergeometric test was applied for KEGG pathway enrichment analysis of these gene families, and *P*-values were adjusted for false discovery rate (FDR) using the *Q*-values method in the R package (https://github.com/StoreyLab/qvalue).

### KS density map and genomic collinearity

We generated a KS density map by calculating the synonymous substitution number for each synonymous site in the collinear genomes of eight plant species. To estimate the timing of polyploidy and speciation events, we applied corrections to the KS density map. These corrections helped clarify whether the duplications observed in different species are shared events, with the process beginning with the earliest known duplication.

Using the *V. vinifera* genome as a reference, we performed colinear gene comparisons with seven other species. Each alignment revealed two peaks. The first peak represents the differentiation event, and the second peak corresponds to the shared duplication event, with the *V. vinifera* genome serving as the baseline for comparison. Since *V. vinifera* has undergone only a single ancient WGT event, its genome was used as the reference. The genomes of *A. graveolens*, *A. sinensis*, *C. sativum*, and *D. carota* were further divided into four sub-genomes, reflecting additional WGD events that took place after their divergence from *V. vinifera*. The *L. sativa* genome was divided into three sub-genomes due to an extra WGT event after its divergence from *V. vinifera*. Similarly, *I. polyneura* and *P. stipuleanatus* genomes were each divided into two sub-genomes due to additional WGD events that occurred post-divergence from *V. vinifera*.

### Extraction and determination of carotenoids

Carotenoids were extracted following a previously described protocol with slight modifications [28]. Leaf, petiole, stem, root, and callus tissue samples from carrot plants were ground into a fine powder and extracted using acetone. The resulting extracts were filtered and analyzed using UPLC on a Shimadzu LC-20 AD system (Japan) equipped with an YMC Carotenoid column (CT99S05-2546WT). Carotenoids, including α-carotene, β-carotene, and lycopene, were identified by comparing them with standard compounds (Supplementary Figs S4 and S7). Carotenoid measurements in overexpression lines were conducted using the Waters e2695 + 2998PDA system with an Agilent LC-C18 column (4.6 × 250 mm, 5 μm) (Supplementary Fig. S6).

### Gene expression analysis

Total RNA was extracted from carrot tissues using the RNA extraction kit (Tiangen, DP441), and cDNA synthesis was performed using the PrimeScript IV First-strand cDNA Synthesis mix (Takara, 6215A). qRT-PCR was carried out with the SYBR™ Green PCR master mix (Thermo Scientific, CAT# 4368577) on an ABI 7500 system. The expressions of structural genes were normalized to the internal reference gene *DcACTIN*. The primers used for PCR are listed in Supplementary Table S18.

### Generation of transgenic and gene-edited carrot plants

To construct the overexpression vectors for *DcLCYB1* and *DcLCYE*, the full-length cDNA sequences of these genes were isolated from ‘Kurodagosun’ and cloned into the pCAMBIA3301 vector. For gene editing, four target sites from each gene were identified using the CRISPR online tool (https://crispor.gi.ucsc.edu/). The corresponding target sequences, driven by the *AtU6–26* and *AtU6–29* promoters and coupled with single guide RNA (sgRNA), were cloned into the gene-editing vector pBSE401 [65]. The resulting recombinant vectors were subsequently transferred into *A. tumefaciens*. Recombinant plasmids were introduced into *A. tumefaciens*, which was then used to infect hypocotyl segments of TXH4 and ‘Kurodagosun’ via a 30-minute co-cultivation approach. The infected hypocotyls were first dedifferentiated on MS medium supplemented with 6-benzyladenine, 2,4-dichlorophenoxyacetic acid, and hygromycin, then redifferentiated on MS medium containing 6-benzyladenine and hygromycin under dark conditions. The regenerated embryonic buds were then transferred to light conditions to stimulate their development into seedlings. Positive transgenic seedlings were transplanted into the soil, and their fleshy roots were harvested after three months. PCR and sequencing were used to validate the transgenic plants and callus tissue. T0 generation transgenic carrot plants and callus tissue were subsequently used for functional analyses. The primer sequences are listed in Supplementary Table S18.

## Supplementary Material

Web_Material_uhaf192

## Data Availability

The TXH4 genome assembly data are accessible in the China National Center for Bioinformation database https://ngdc.cncb.ac.cn/gwh/ (CNA0423801).
