# Supplementary material for: Analysis of telomere-to-telomere genome of red carrot TXH4 elucidates the role of DcLCYE and DcLCYB1 in lycopene accumulation in carrot
Source: Hortic Res. 2025 Jul 29;12(11):uhaf192. doi: 10.1093/hr/uhaf192 (PMC12552772; doi:10.1093/hr/uhaf192)
Supplement: Web_Material_uhaf192 [file web_material_uhaf192.zip › 20250708 Supplementary material R2.docx]

**Analysis of telomere-to-telomere genome of red carrot TXH4 elucidates the role of DcLCYE and DcLCYB1 in lycopene accumulation in carrot**

**Supplementary material**

**
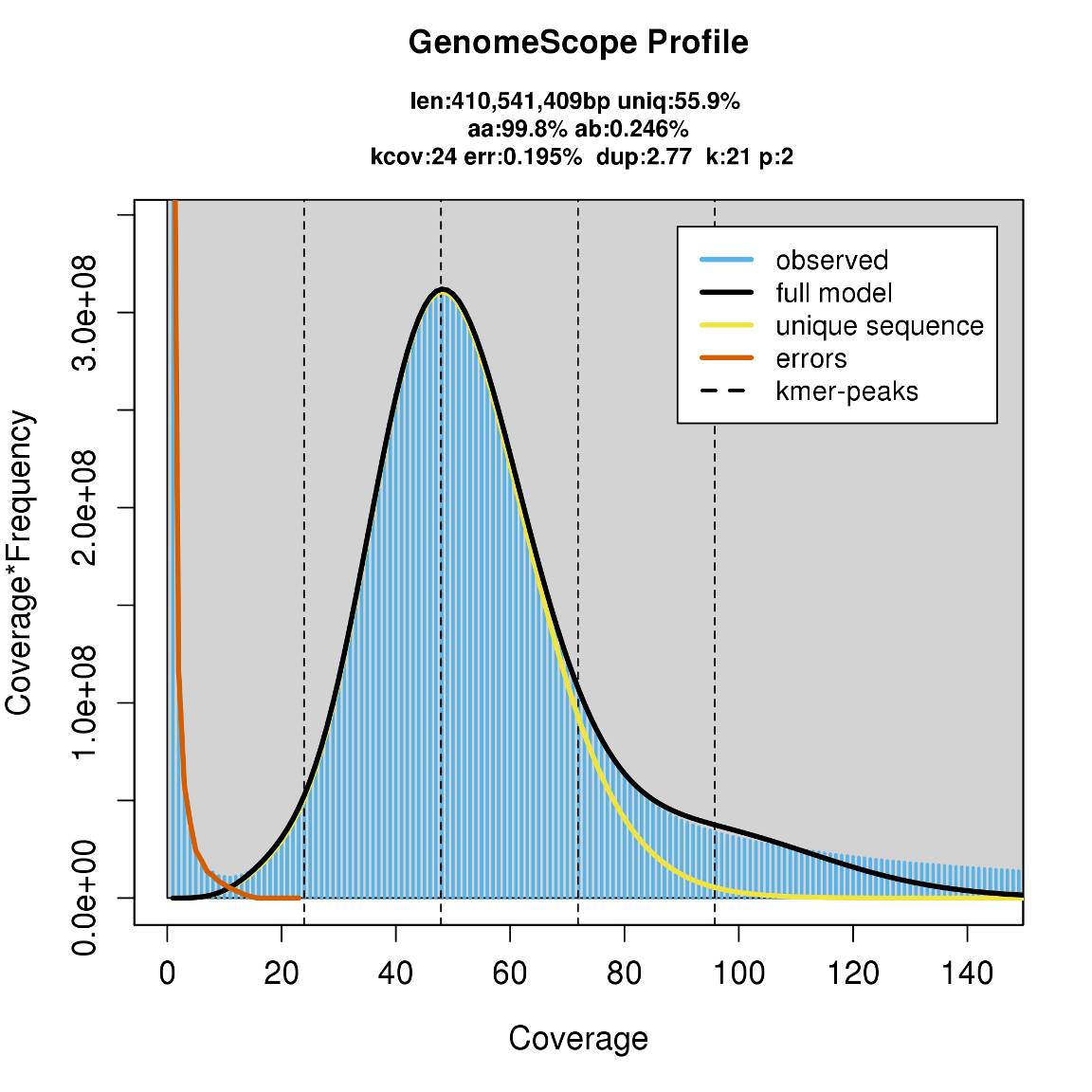
**

**Figure S1. GenomeScope profile analysis of the target genome.** The plot displays the k-mer frequency distribution (blue bars) and the GenomeScope model fit (black line) for the genome assembly. The yellow line represents unique sequences, while the orange line highlights sequencing errors. The dashed vertical lines indicate k-mer peaks at varying coverage levels.


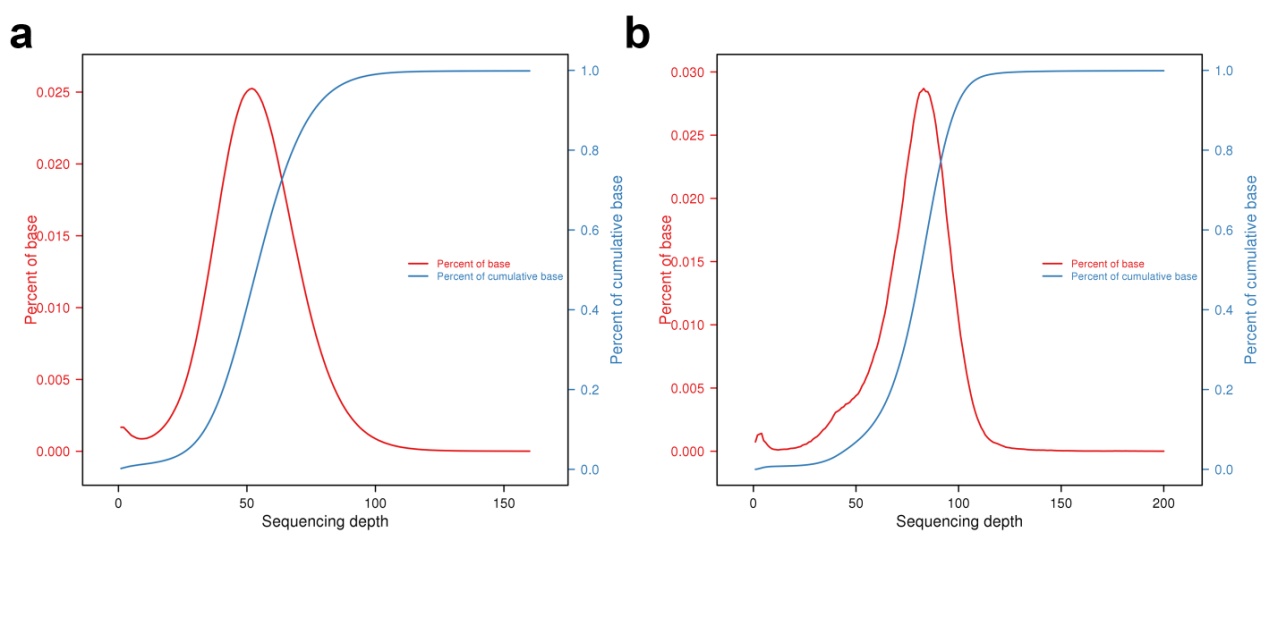


**Figure S2. Depth distribution for Illumina sequencing (a) and HiFi sequencing (b).** The red line represents the percentage of bases at each sequencing depth, while the blue line shows the cumulative percentage of bases.


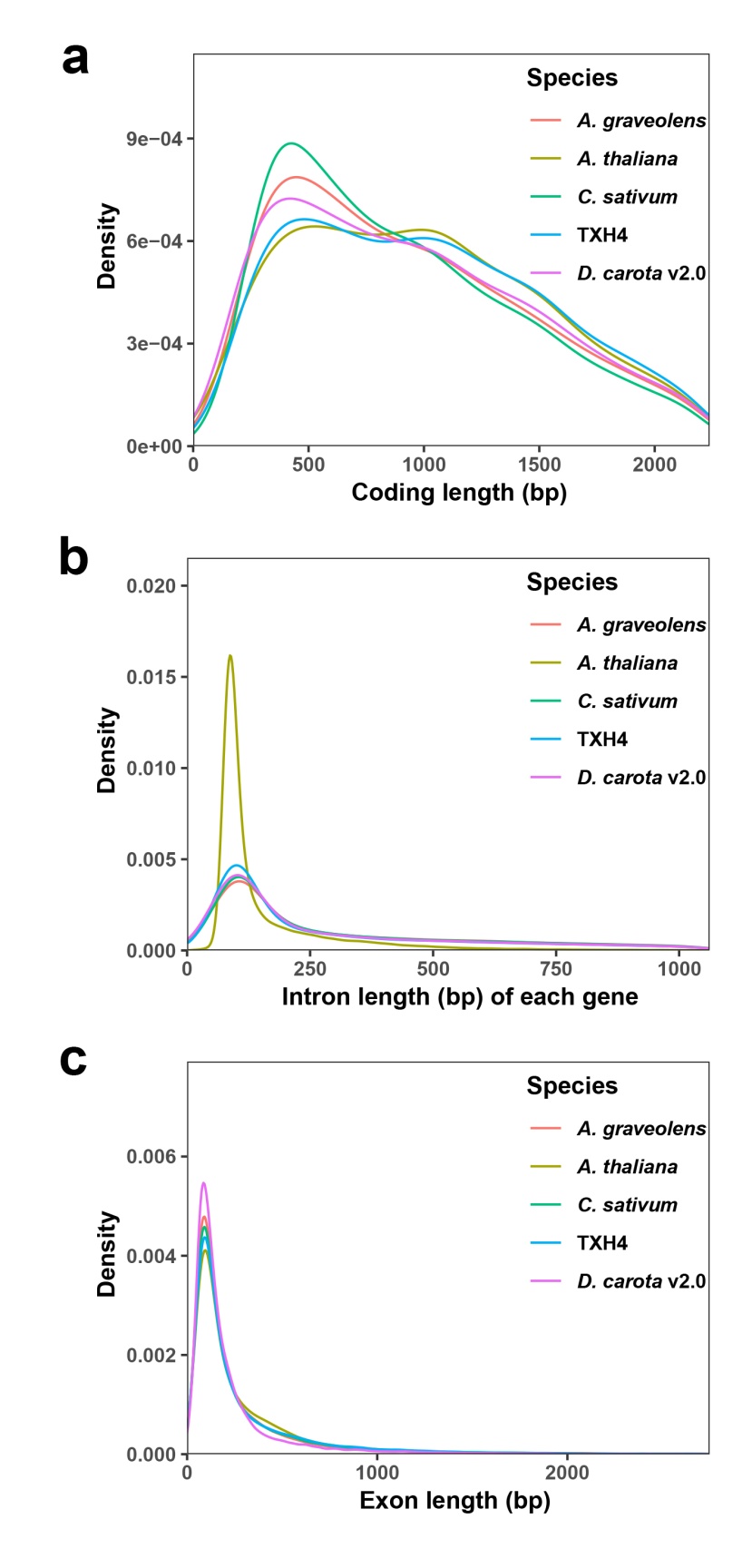


**Figure S3. The length distribution of key gene structure components, such as coding regions (a), introns (b), and exons (c) in the T2T genome of carrot TXH4 by comparing it with those of *A. graveolens*, *A. thaliana*, *C. sativum*, and *D. carota* v2.0.**


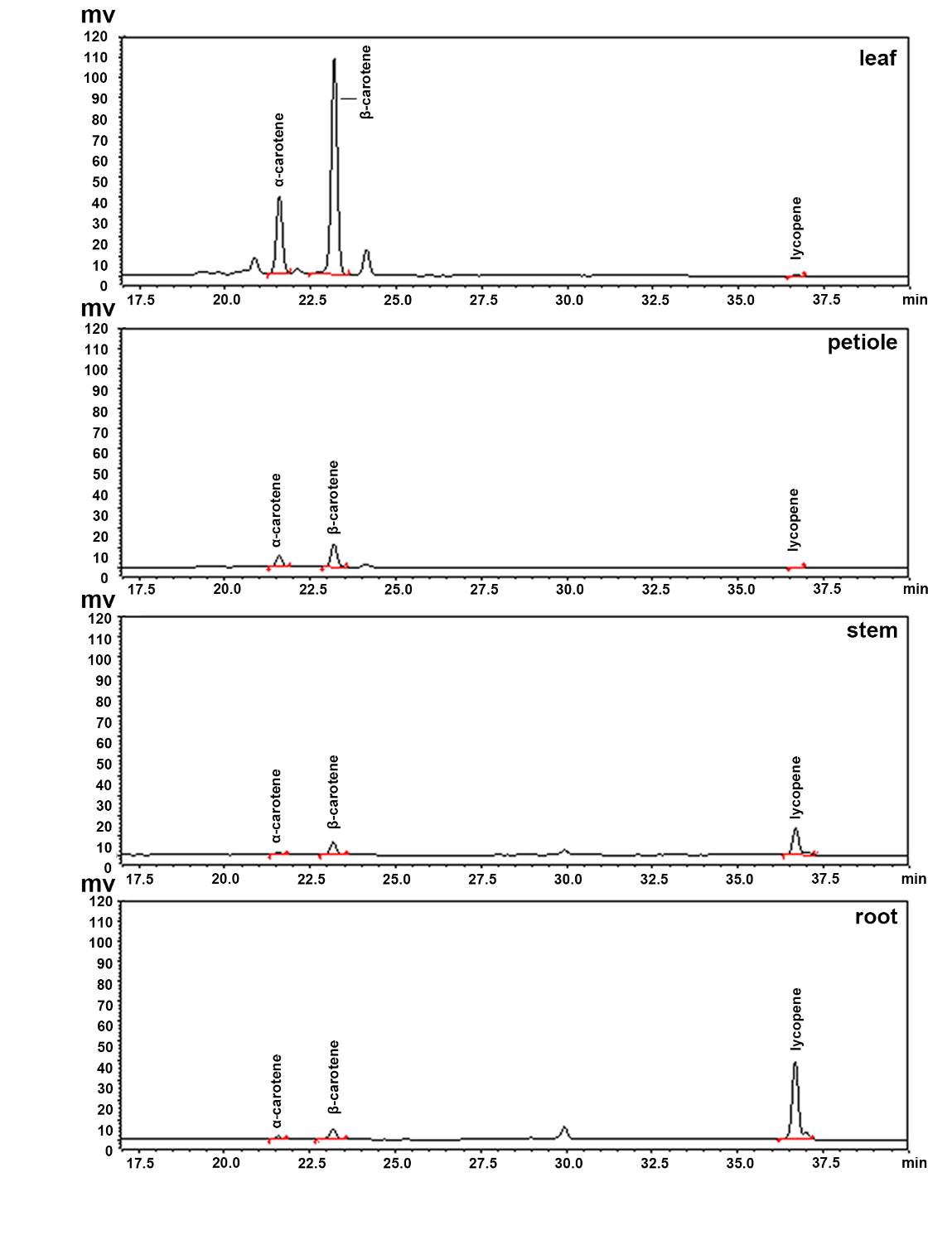


**Figure S4. Ultra-performance liquid chromatography (UPLC) chromatograms for carotenoid analysis in the leaf, petiole, stem, and root tissues of TXH4.**

**
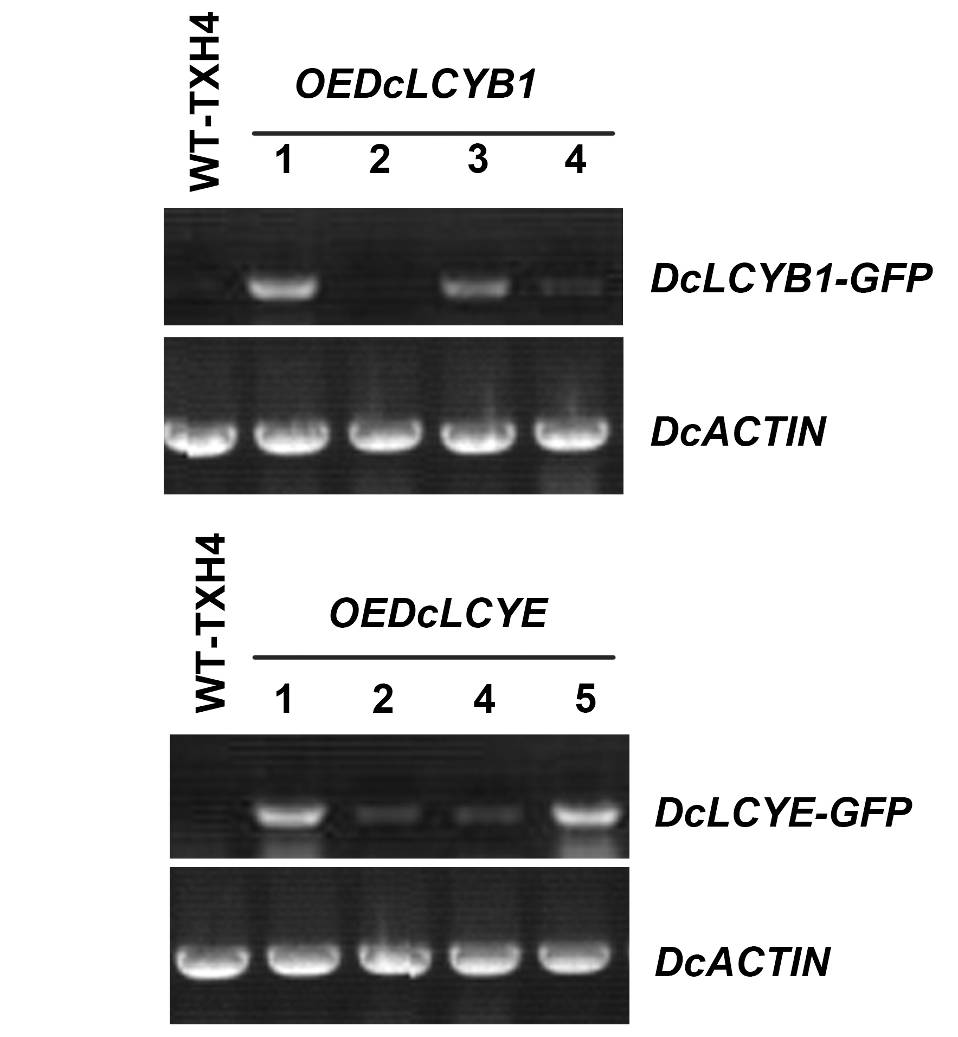
**

**Figure S5. *DcLCYB1-GFP* (a) and *DcLCYE-GFP* (b) expressions in carrot overexpression lines with *DcACTIN* used as an internal reference.**

**
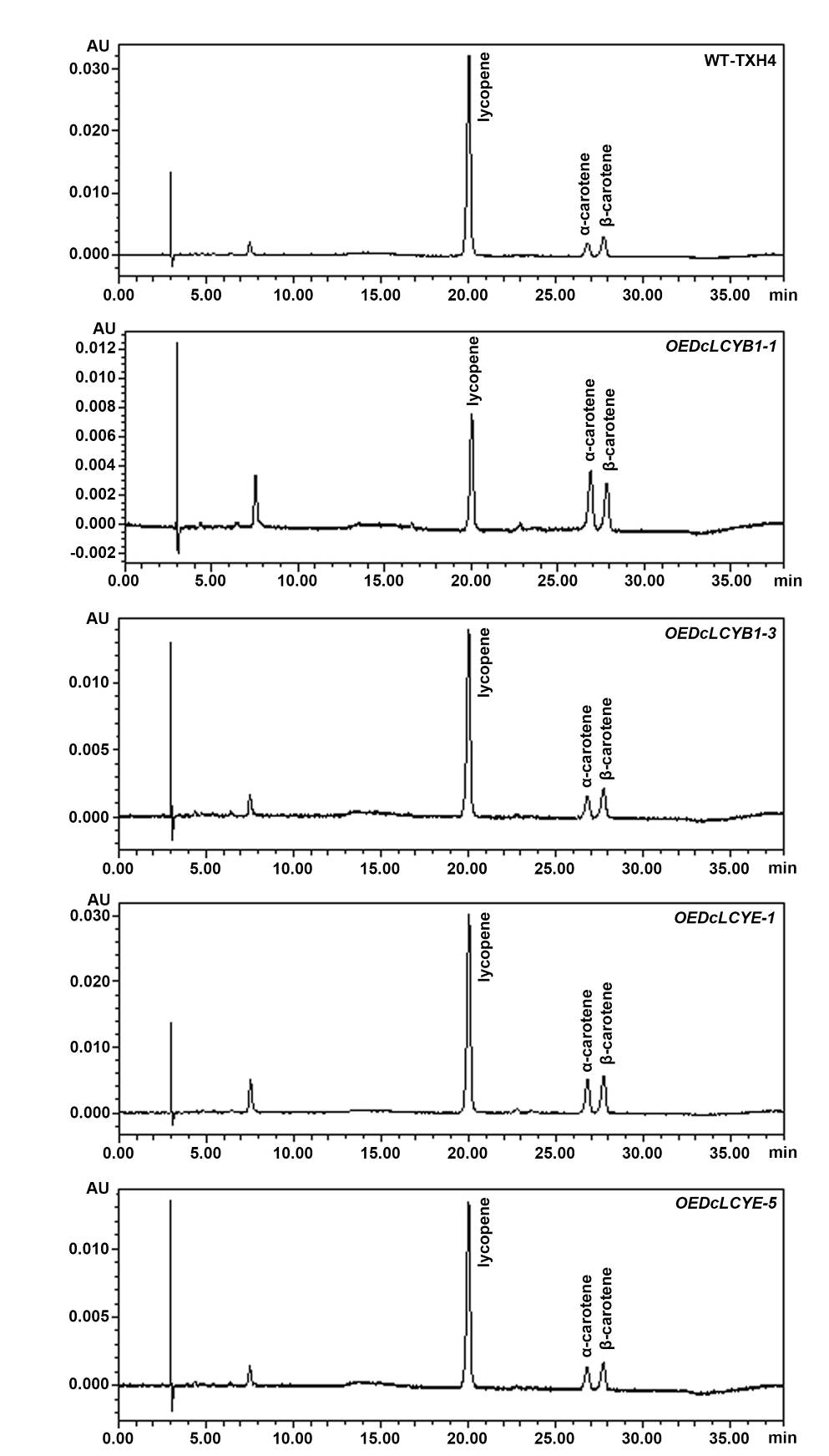
**

**Figure S6. UPLC chromatograms depicting the carotenoid profiles of *DcLCYB1* and *DcLCYE* overexpression plants.**

**
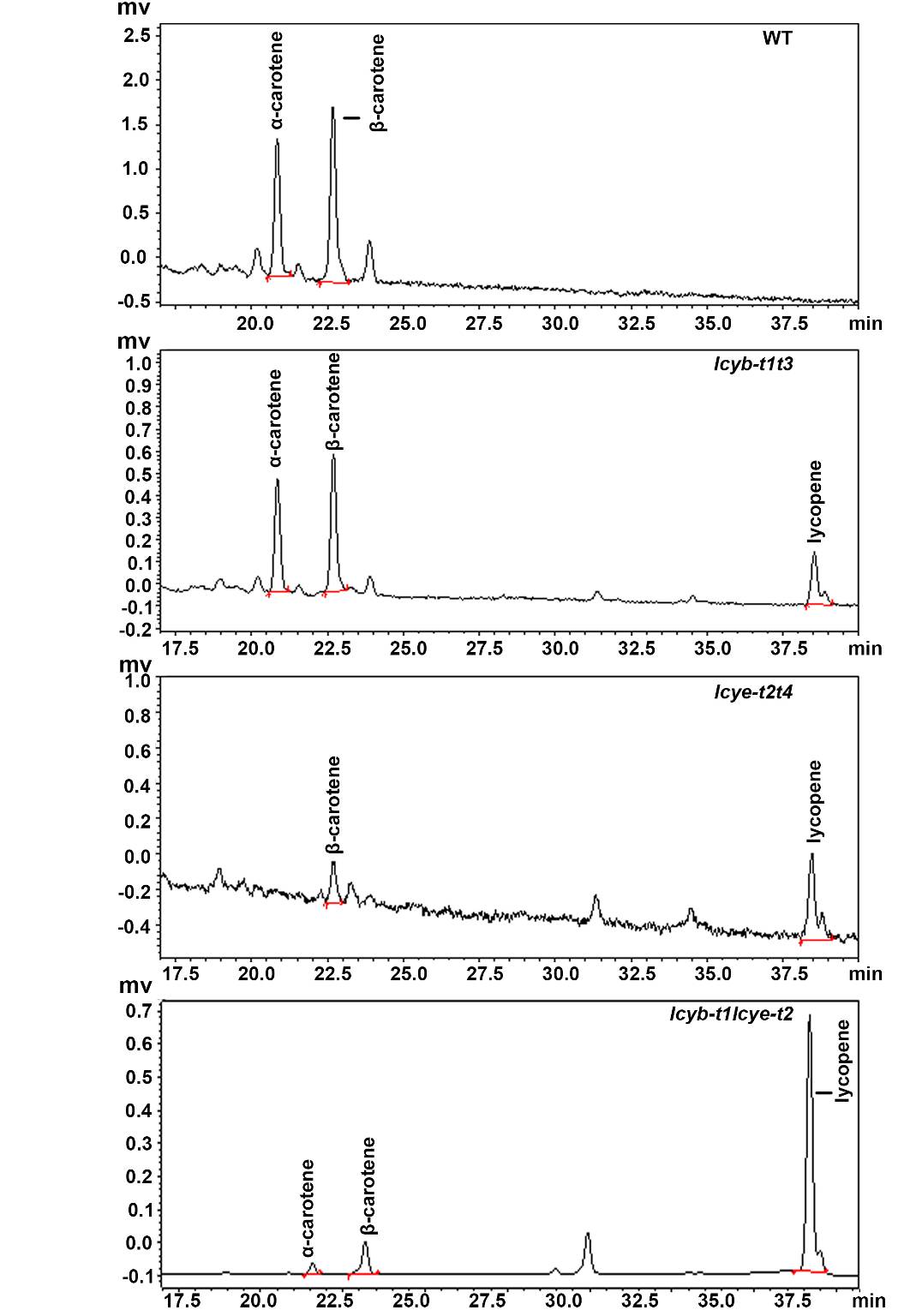
**

**Figure S7. UPLC chromatograms showing carotenoid profiles in callus tissue after *DcLCYB1* and *DcLCYE* knockout.**

**Table S1. Assembly statistics of the TXH4 genome.**

**Table S2. Summary of key statistics from the CEGMA and BUSCO analyses.**

**Table S3. Summary of the distribution and composition of transposable elements identified in the TXH4 genome.**

**Table S4. The detailed analysis and distribution of satellite sequences within the TXH4 genome.**

**Table S5. The number of genes in TXH4 predicted using various methods.**

**Table S6. Comparative analysis of gene structure characteristics among the genomes of *A. thaliana*, *C. sativum*, *D. carota* v2.0, *A. graveolens*, and TXH4.**

**Table S7. The gene annotation of the TXH4 genome across various protein databases.**

**Table S8. The number and lengths of pseudogenes identified in the TXH4 genome.**

**Table S9. Count of non-coding RNAs in the TXH4 genome.**

**Table S10. Statistics of protein-coding genes across nine species: *A. graveolens*, *A. sinensis*, *A. thaliana*, *C. sativum*, *D*. *carota*, *I. polyneura*, *L. sativa*, *P. stipuleanatus*, and *V. vinifera.***

**Table S11. Summary of counts, lengths, and structure of SNPs in *D. carota* T2T and *D. carota* v2.0 relative to the TXH4 genome.**

**Table S12.** **Summary of counts, lengths, and structure of InDels in *D. carota* T2T and *D. carota* v2.0 relative to the TXH4 genome.**

**Table S13. Structural rearrangements in *D. carota* T2T and *D. carota* v2.0 relative to the TXH4 genome.**

**Table S14. Summary of counts and lengths of presence and absence variations specific to the *D. carota* T2T and *D. carota* v2.0 relative to the TXH4 genome.**

**Table S15. Primers used in the experiments in this study.**
