# Supplementary figures and images for: Analysis of telomere-to-telomere genome of red carrot TXH4 elucidates the role of DcLCYE and DcLCYB1 in lycopene accumulation in carrot
Source: Hortic Res. 2025 Jul 29;12(11):uhaf192. doi: 10.1093/hr/uhaf192 (PMC12552772; doi:10.1093/hr/uhaf192)

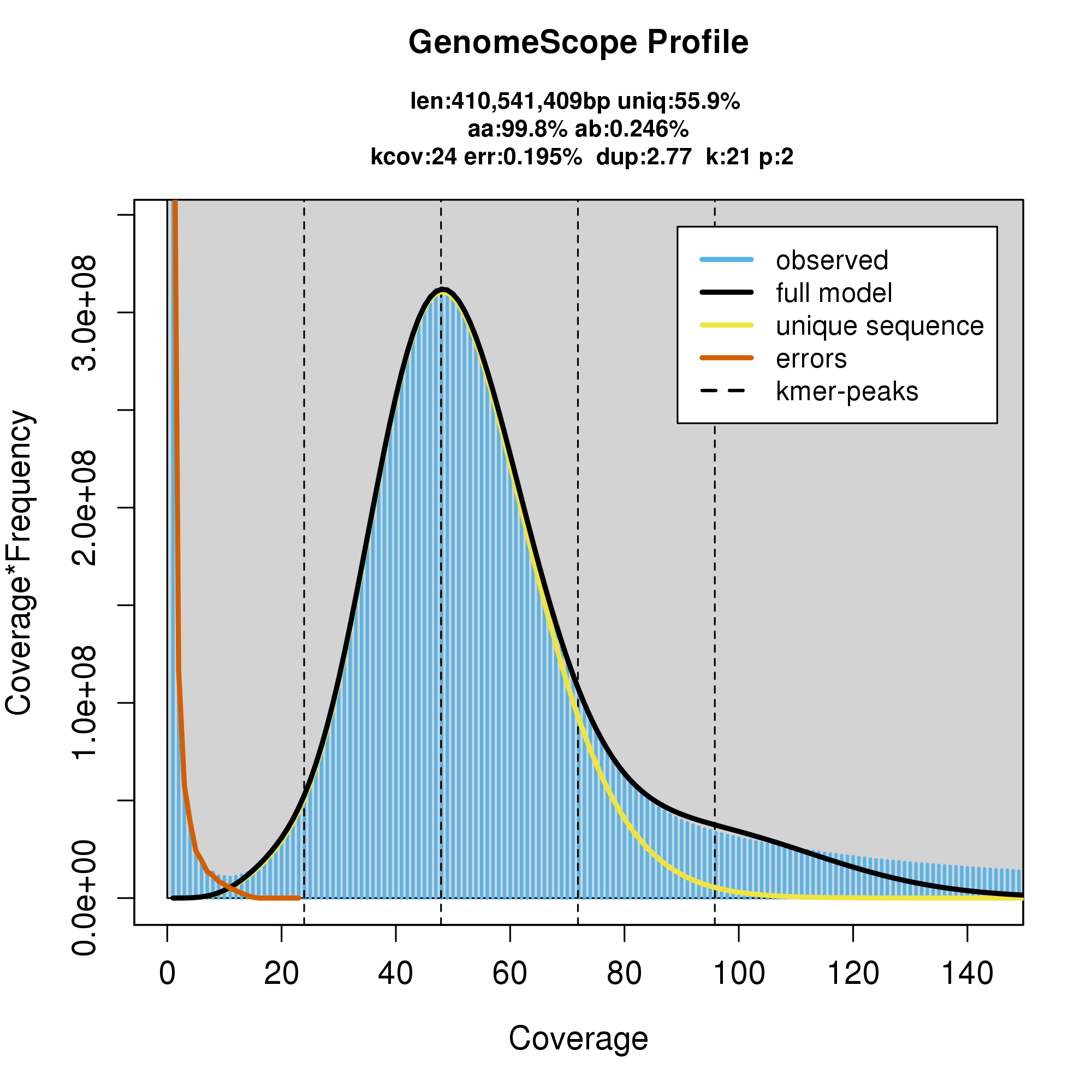

Supplement: Web_Material_uhaf192 [file web_material_uhaf192.zip › Fig S1.jpg]

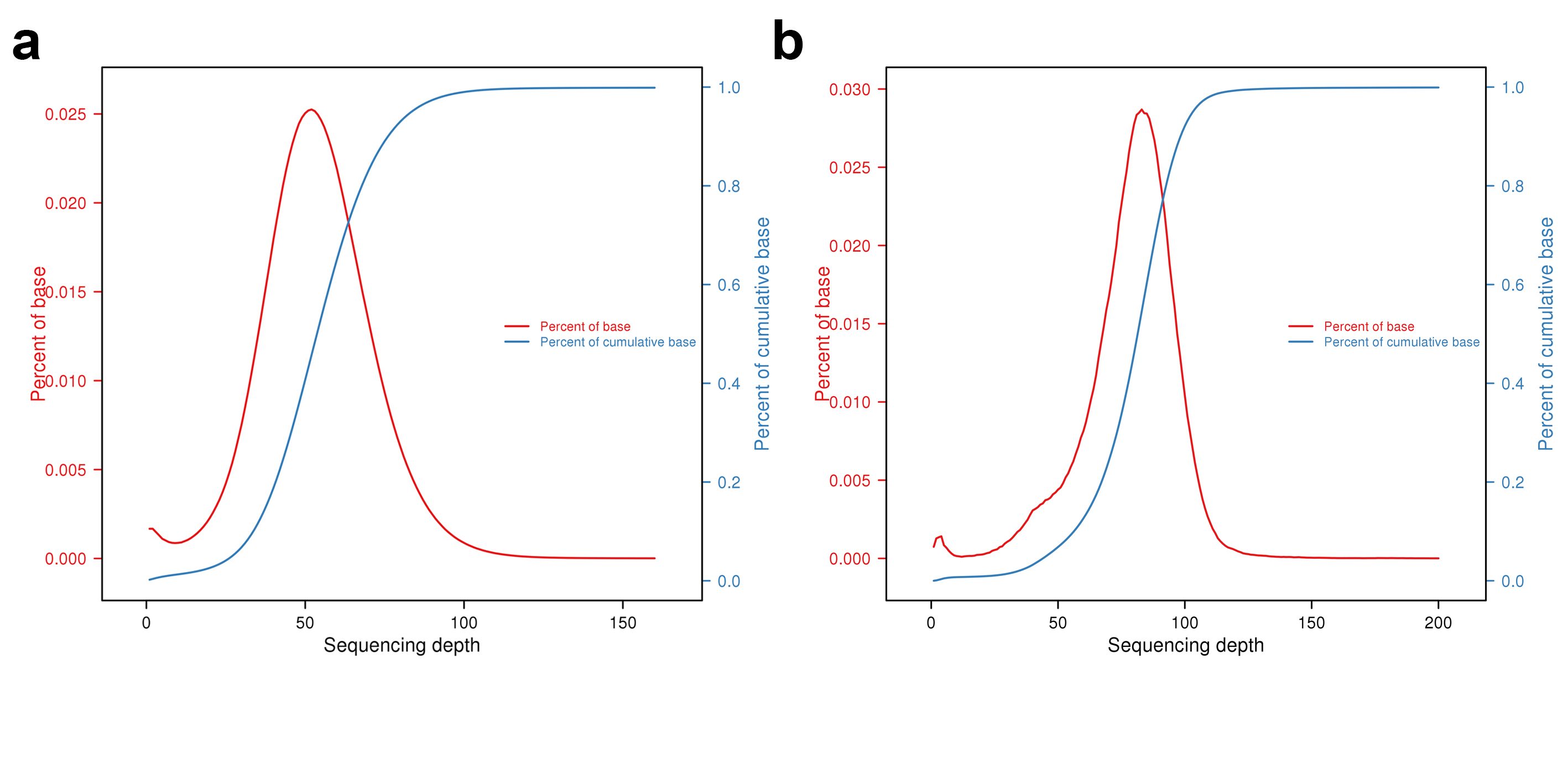

Supplement: Web_Material_uhaf192 [file web_material_uhaf192.zip › Fig S2.jpg]

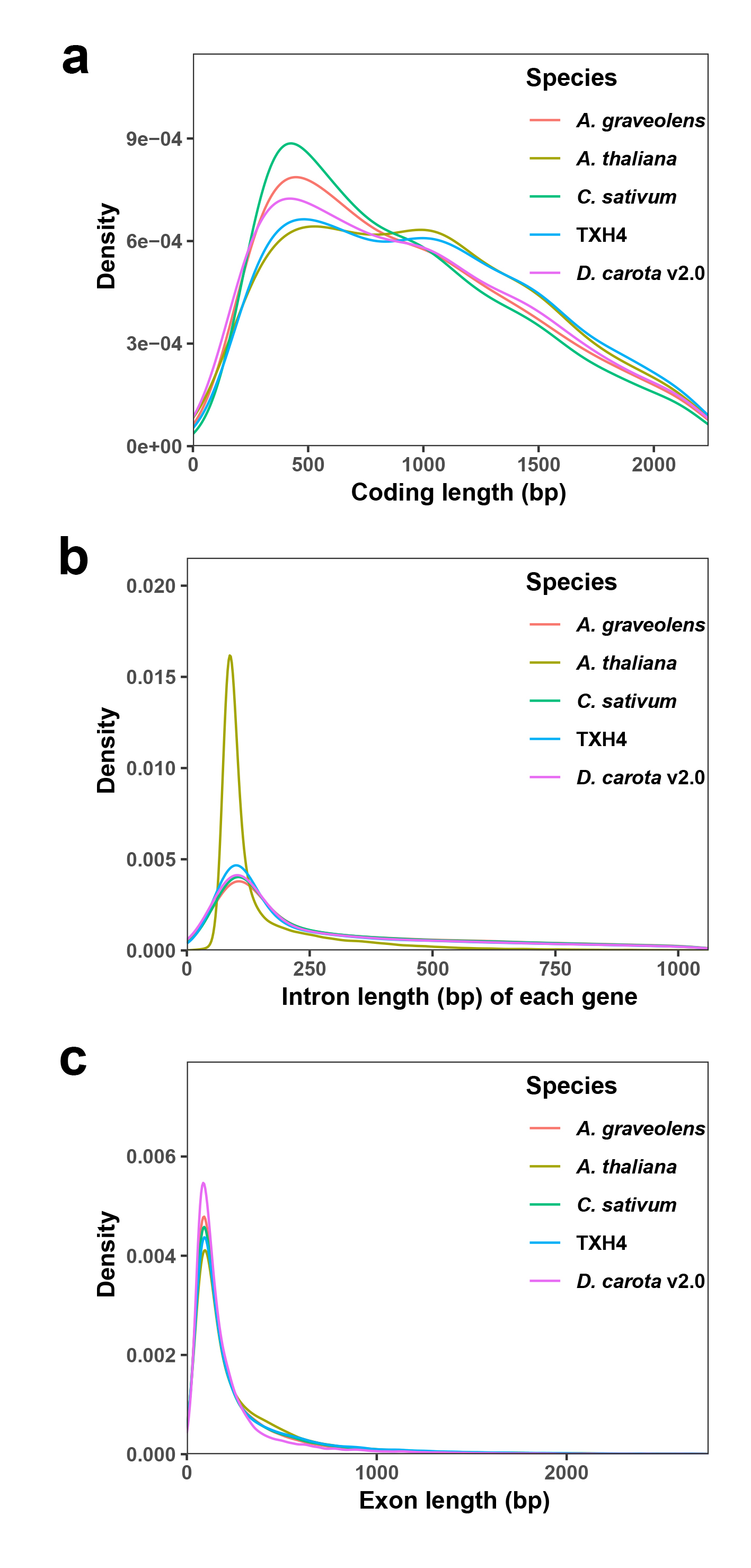

Supplement: Web_Material_uhaf192 [file web_material_uhaf192.zip › Fig S3.jpg]

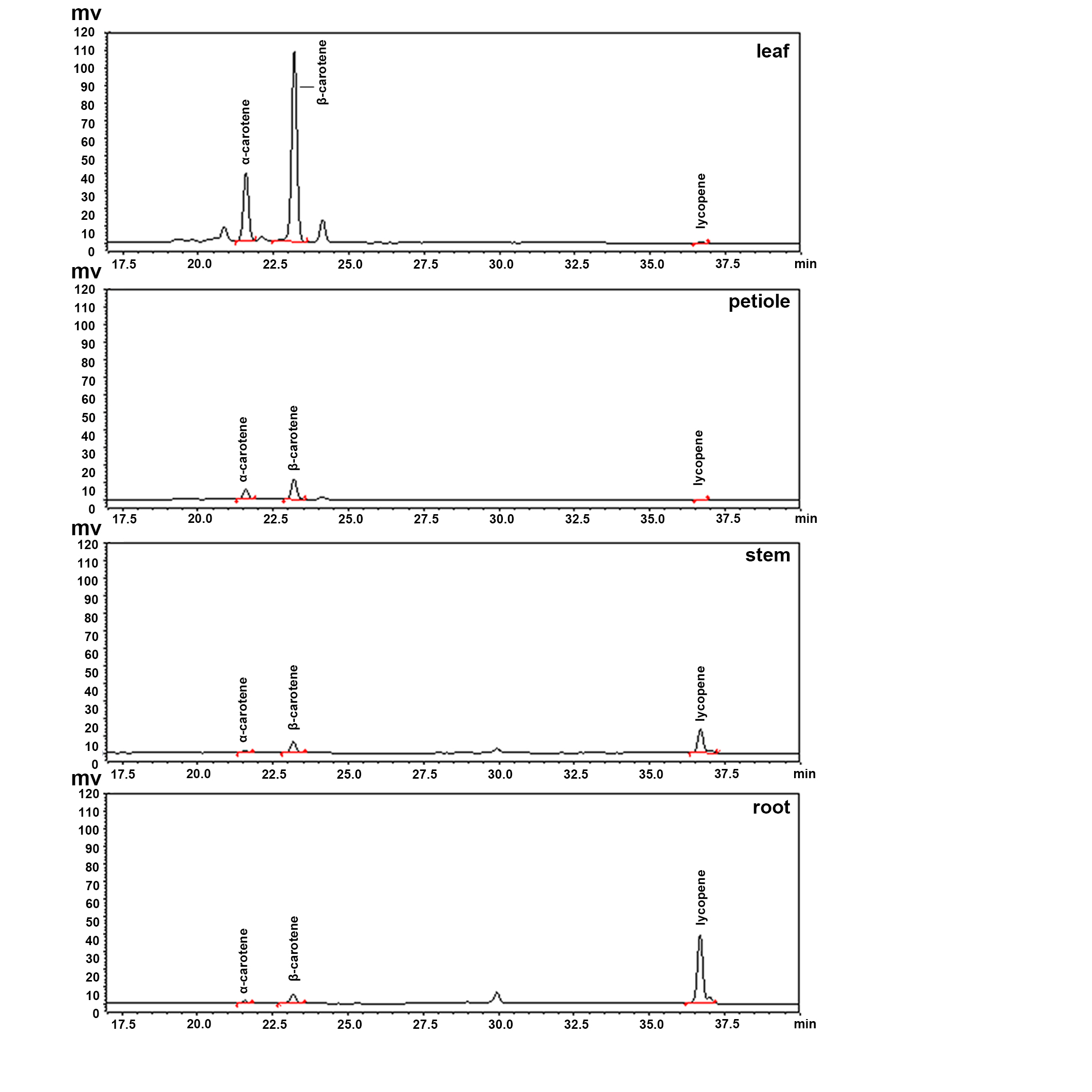

Supplement: Web_Material_uhaf192 [file web_material_uhaf192.zip › Fig S4.jpg]

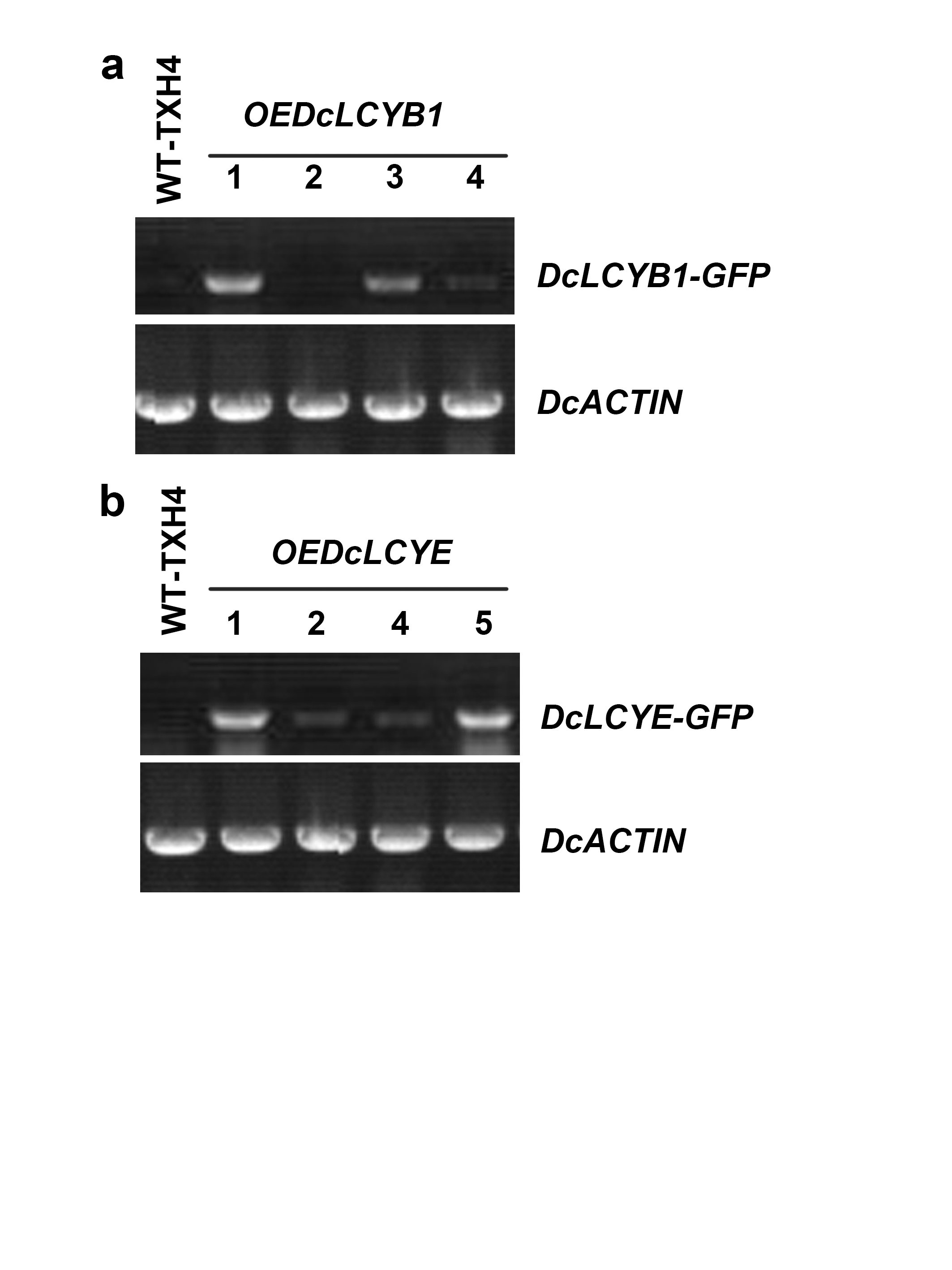

Supplement: Web_Material_uhaf192 [file web_material_uhaf192.zip › Fig S5.jpg]

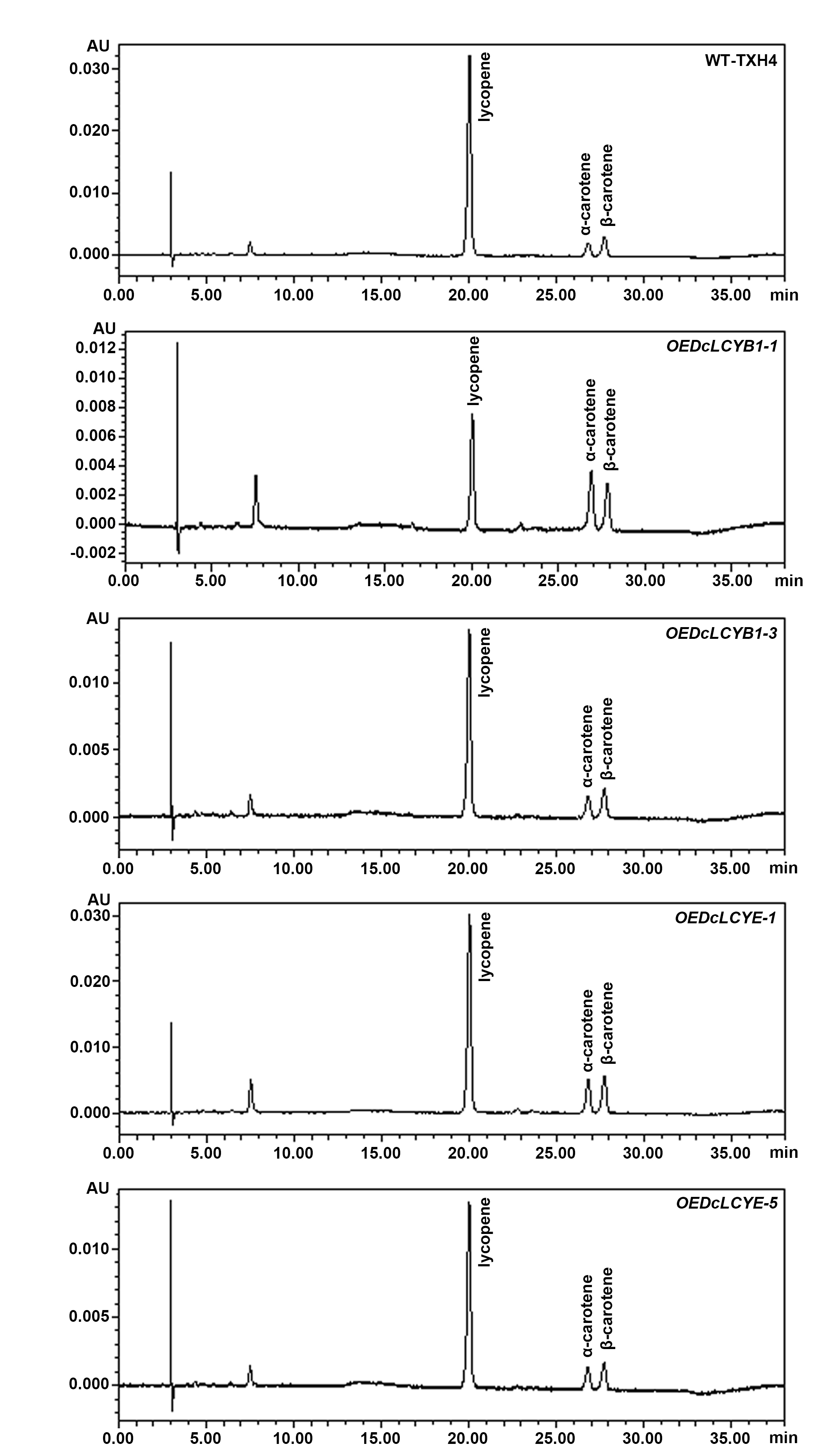

Supplement: Web_Material_uhaf192 [file web_material_uhaf192.zip › Fig S6.jpg]

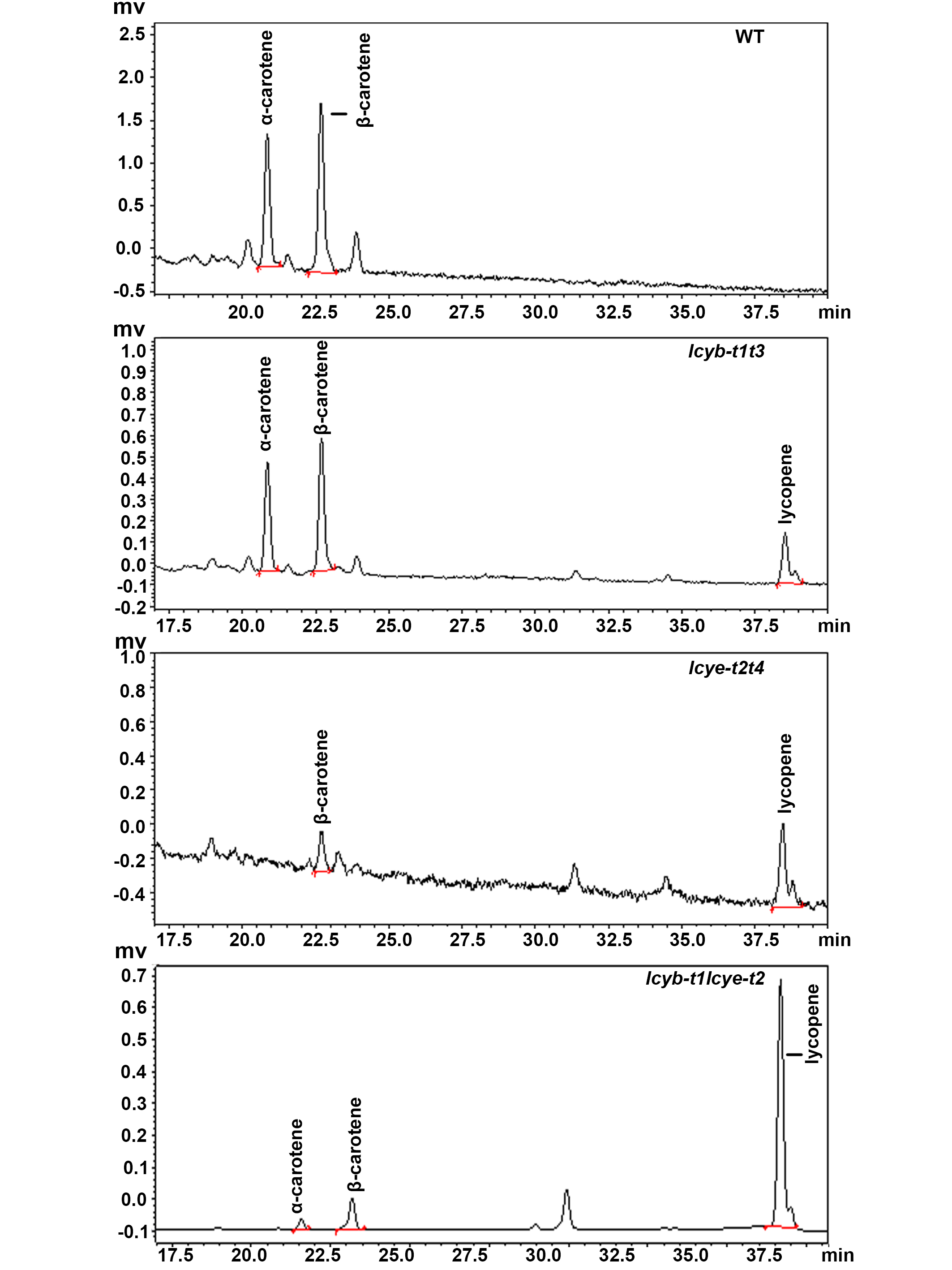

Supplement: Web_Material_uhaf192 [file web_material_uhaf192.zip › Fig S7.jpg]
